# Synthesis, Characterization, and Electrochemistry of Diferrocenyl β-Diketones, -Diketonates, and Pyrazoles †

**DOI:** 10.3390/molecules25194476

**Published:** 2020-09-29

**Authors:** Steve W. Lehrich, Julia Mahrholdt, Marcus Korb, Alexander Hildebrandt, Jannie C. Swarts, Heinrich Lang

**Affiliations:** 1 Anorganische Chemie, Institut für Chemie, Fakultät für Naturwissenschaften, Technische Universität Chemnitz, D-09107 Chemnitz, Germany; steve-lehrich@web.de (S.W.L.); julia.mahrholdt@chemie.tu-chemnitz.de (J.M.); alexander.hildebrandt@chemie.tu-chemnitz.de (A.H.); 2 School of Molecular Sciences, The University of Western Australia, 35 Stirling Highway, Crawley, Perth, WA 6009, Australia; marcus.korb@uwa.edu.au; 3 Department of Chemistry, University of the Free State, P.O. Box 339, Bloemfontein 9300, South Africa; swartsjc@ufs.ac.za

**Keywords:** ferrocenyl, pyrazole, β-diketonate, solid-state structure, spectro(electrochemistry), electron transfer

## Abstract

The synthesis of FcC(O)CH(R)C(O)Fc (Fc = Fe(η^5^-C_5_H_4_)(η^5^-C_5_H_5_); R = H, **5**; *^n^*Bu, **7**; CH_2_CH_2_(OCH_2_CH_2_)_2_OMe, **9**), [M(κ^2^*O*,*O*′-FcC(O)CHC(O)Fc)_n_] (M = Ti, *n* = 3, **10**; M = Fe, *n* = 3, **11**; M = BF_2_, *n* = 1, **12**), and 1-R′-3,5-Fc_2_-*^c^*C_3_HN_2_ (R′ = H, **13**; Me, **14**; Ph, **15**) is discussed. The solid-state structures of **5**, **7**, **9**, **12**, **13**, **15**, and **16** ([TiCl_2_(κ^2^*O*,*O*′-PhC(O)CHC(O)Ph)_2_]) show that **7** and **9** exist in their β-diketo form. Compound **13** crystallizes as a tetramer based on a hydrogen bond pattern, including one central water molecule. The electrochemical behavior of **5**–**7** and **9**–**16** was studied by cyclic and square-wave voltammetry, showing that the ferrocenyls can separately be oxidized reversibly between −50 and 750 mV (**5**–**7**, **9**, **12**–**15**: two Fc-related events; **10**, **11**: six events, being partially superimposed). For complex **10**, Ti-centered reversible redox processes appear at −985 (Ti^II^/Ti^III^) and −520 mV (Ti^III^/Ti^IV^). Spectro-electrochemical UV-Vis/NIR measurements were carried out on **5**, **6,** and **12**, whereby only **12** showed an IVCT (intervalence charge-transfer) band of considerable strength (*ν_max_* = 6250 cm^−1^, *Δν_½_* = 4725 cm^−1^, *ε_max_* = 240 L·mol^−1^·cm^−1^), due to the rigid C_3_O_2_B cycle, enlarging the coupling strength between the Fc groups.

## 1. Introduction

Main-group element and transition metal β-diketonates, including Lewis-base adducts thereof, belong to one of the most investigated metal-organic complexes [1,2,3,4,5,6,7]. One reason is that they can be prepared by straightforward synthetic methodologies [8,9,10], and that many of them are commercially available. Hence, they attained great interest in, for example, the deposition of metal or metal oxide thin layers by applying diverse deposition techniques, including CVD (=chemical vapor deposition) [1,11,12,13,14], ALD (=atomic layer deposition) [15,16,17,18,19], and spin-coating [20,21,22]. In addition, they received great interest in the synthesis of (hetero)multimetallic complexes [23,24,25,26,27,28,29] as catalysts in organic synthesis [30,31,32] and as anticancer drugs (especially copper and rhodium β-diketonates) [33,34,35]. Recently, they gained topical attention in the study of electron transfer processes, when, for example, redox-active groups such as ferrocenyls are present, i.e., in zirconium, hafnium, copper(I), copper(II), manganese, ruthenium, rhodium(I), and aluminum β-diketonate complexes [36,37,38,39,40,41].

Pyrazoles are a major class of heterocyclic compounds and have been thoroughly studied [42,43,44,45,46,47]. They show, for example, biological activities possessing antimicrobial, anti-inflammatory, or antitumor properties [48,49,50,51,52,53,54]. In this respect, it was found that ferrocenyl-functionalization of pyrazoles enhances their biological activities [55,56,57,58,59]. Facile synthesis methods of ferrocenyl-substituted pyrazoles were reported [60,61,62,63,64,65,66], with only a brief comment about the parent diferrocenyl NH-pyrazole in 1963 [66].

Against these backgrounds, we recently became interested in the synthesis of ferrocenyl β-diketones and on their complexation behavior towards transition metal and main-group element building blocks as well as in the preparation of diferrocenyl pyrazoles to study their electrochemical and spectro-electrochemical properties. It is a continuation of recently published work from our group in the field of ferrocenyl-substituted carbon-rich π-conjugated systems [67,68,69,70,71,72,73,74,75,76,77,78].

We herein enrich the families of diferrocenyl β-diketones, β-diketonates, and pyrazoles by the synthesis of FcC(O)CH(R)C(O)Fc, [M(κ^2^*O*,*O*′-FcC(O)CHC(O)Fc)_n_], and 1-R′-3,5-Fc_2_-*^c^*C_3_HN_2_, respectively, (Fc = Fe(η^5^-C_5_H_4_)(η ^5^-C_5_H_5_); R = H, CH_2_CH_2_(OCH_2_CH_2_)_2_OMe; R′ = H, Me, Ph; M = Ti, *n* = 3; M = Fe, *n* = 3; M = BF_2_, *n* = 1). Their molecular solid-state structures and (spectro)electrochemical behavior are reported as well.

## 2. Results and Discussion

### 2.1. Synthesis and Characterization

**Scheme 1:**
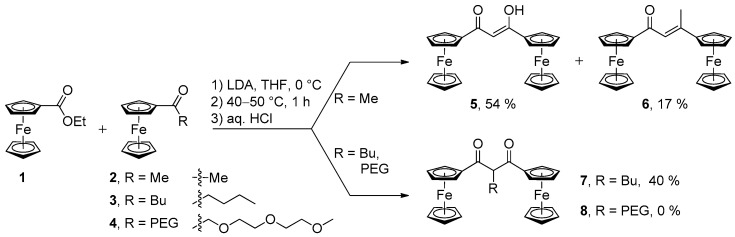
Synthesis of **5**–**8** (LDA = LiN*^i^*Pr_2_; THF = tetrahydrofuran; yields are based on ferrocene **1**).

The synthetic methodologies to prepare the ferrocenyl-functionalized β-diketones **5**–**8** is shown in Scheme 1. The therefore necessary starting compounds **1**–**3** were synthesized accordingly to references [79,80,81]. Ketone **4** was prepared by a Friedel-Crafts acylation of ferrocene with 2-[2-(2-methyoxyethoxy)ethoxy]acetyl chloride, which is accessible by refluxing the appropriate carboxylic acid with thionyl chloride in analogy to references [82,83], with ferrocene in presence of the Lewis acid catalyst AlCl_3_ (Section 4.6). 

Claisen condensation of **1** with **2** resulted in the formation of diferrocenyl β-diketone **5**. Side product **6** could be isolated (Scheme 1) [84], which was produced by self-aldol condensation of acetylferrocene (**2**). Using KO*^t^*Bu (DMF, 50 °C) as the base instead of LDA (=lithium diisopropylamide) minimized the yield of side product **6** to 7%, while slightly increasing the amount of **5** (58%).

**Scheme 2:**
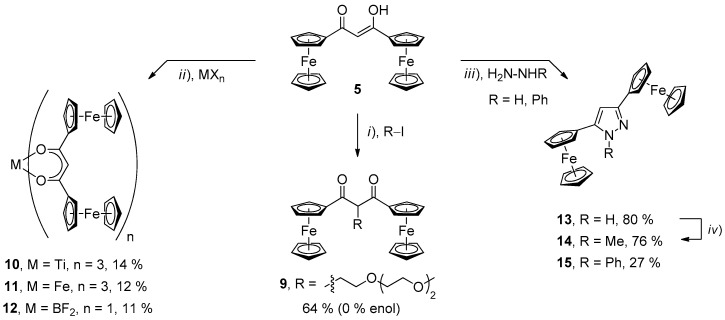
Synthesis of **9** (*i*), **10**–**12** (*ii*), and **13**–**15** (*iii*). (*i*) KO*^t^*Bu, 1-iodo-2-[2-(2-methoxyethoxy)ethoxy]ethane, DMSO, 50 °C, 14 h; (*ii*) **10**: TiCl_4_, THF, −80 °C to 25 °C; **11**: [Fe(acac)_3_], reflux, 3 h; **12**: [BF_3_∙Et_2_O], HN*^i^*Pr_2_, ambient temperature, 1 h; (*iii*) NH_2_-NHR, AcOH, 70 °C, 12 h; (*iv*) NEt*^i^*Pr_2_, PhNO_2_, Me_2_SO_4_, 70 °C, 12 h (DMSO=dimethyl sulfoxide).

To improve the solubility of the respective diferrocenyl β-diketones and the appropriate metal complexes (Scheme 1 and Scheme 2), a butyl and ethylene glycol chain, respectively, was introduced in β-position of the β-diketone. Thus, Claisen condensation of **1** with **3** yielded **7**, whereas treatment of **1** with **4** to give **8** was not successful. 

Thus, a different approach was applied to synthesize an ethylene glycol functionalized diferrocenyl β-diketone. Hence, compound **5** was treated with 1-iodo-2-[2-(2-methoxyethoxy)ethoxy]ethane and KO*^t^*Bu at 50 °C (pathway *i*), Scheme 2 whereby ICH_2_CH_2_(OCH_2_CH_2_)_2_OMe was prepared by applying the Finkelstein reaction (treatment of BrCH_2_CH_2_(OCH_2_CH_2_)_2_OMe with NaI in acetone [85,86]. The respective BrCH_2_CH_2_(OCH_2_CH_2_)_2_OMe educt was obtained from an Appel reaction starting from 2-[2-(2-methoxyethoxy)ethoxy]ethanol with CBr_4_ and PPh_3_, respectively [87,88].) It should be noted that alkyl-substituted **7** and **9** were obtained in their β-diketo form, whereas **5** contained ~66% of the enol isomer. The value is similar to those reported in literature [84] and was evidenced by the presence of the CH resonance at ~5.9 ppm and a broad signal at ~16.5 ppm of the de-shielded OH functionality.

In addition, diferrocenyl diketone **5** was applied as a starting material for the synthesis of the titanium, iron, and boron β-diketonato coordination complexes **10**–**12** (pathway *ii*), Scheme 2. Therefore, following two synthetic methodologies were used: either ligand exchange [41] or lithium-halide metathesis. In this respect, coordination complex **10** was accessible by the addition of TiCl_4_ to a tetrahydrofuran solution containing [Li(κ^2^*O*,*O*′-FcC(O)CHC(O)Fc)] [41] at −80 °C, and **11** by refluxing [Fe(acac)_3_] (acac = acetylacetonate) with a 3-fold excess of **5** in acetonitrile (Scheme 2). For the preparation of the purple dioxaborine complex **12**, diisopropylamine and [BF_3_∙Et_2_O] were subsequently reacted with **5** at ambient temperature. The low yields of **10**–**12** are similar to the recently synthesized Al complex and can be explained with the steric hindrance of the ferrocenyl-functionalized ligands [41].

Nonetheless, metalation of **7** and **9** by using different reagents such as KO*^t^*Bu, LDA, or *^n^*BuLi between −80 °C to 40 °C in different solvents (THF, hexane) was not successful, which most probably is attributed to the low acidity of the α-hydrogen atom of the β-diketone caused by the electron-rich Fc and alkyl groups. In all of these studies, solely the starting materials were recovered in virtually quantitative yield. This was proven for compound **8** by addition of electrophiles (MeI and Me_2_SO_4_) to the reaction mixture containing a potentially lithiated species of **8**, whereby a methylated compound was not detected. It should be noted that deprotonation of the herein less-acidic α-hydrogen could compete with a metalation of the C_5_H_4_ group, due to the *ortho*-directing properties of the adjacent carbonyl X=O (X = C, P, S) functionalities [89,90,91,92,93,94,95]. However, such species were also not observed upon treatment with the mentioned electrophiles at −80, −40, 0, and 40 °C, which might have two reasons. First, the lithiation rate is insufficient at low temperatures (−80 to −40 °C) and non-polar solvents (hexane) [91,93,94,95]. Second, it is known that lithiated ferrocenes can be re-protonated via ether cleavage of THF or Et_2_O. The rate constant for this reaction accordingly increases at higher temperatures (40 °C), giving the starting material back [96,97,98]. 

A straightforward synthesis procedure for diferrocenyl-functionalized pyrazoles **13**–**15** is given by the reaction of **5** with an excess of hydrazines NH_2_-NHR (R = H, Ph), as outlined in Scheme 2 (pathway *iii*)) (Section 4). 

In case of **15**, however, the higher electron density of the *N*-phenyl group reduced the ability of the intermediate hydrazone to undergo a successful ring-closure. Methyl hydrazine failed to react to give **14** and most of **5** was recovered. Thus, formation of methyl derivative **14**, possessing an even higher electron density, had to be achieved via methylation of the H-analogue **13**.

**Scheme 3:**
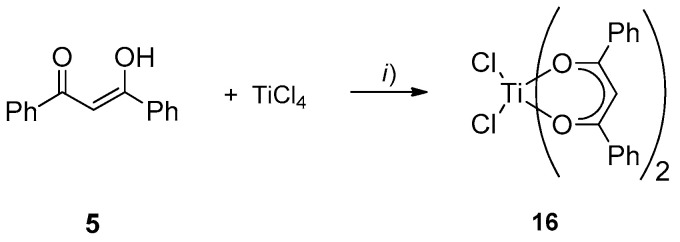
Synthesis of **16** (*i*) THF, −80 °C to 25 °C, 2 h.

For comparable purposes, regarding the discussion of the electrochemical behavior of **10**, we attempted to prepare the isostructural complex [Ti(κ^2^*O*,*O*′-PhC(O)CHC(O)Ph)_3_] [99]. However, it appeared that within the reaction of 1,3-diphenyl-1,3-propanedione with TiCl_4_ solely the corresponding Ti(IV) coordination complex **16** was produced (Scheme 3) [100,101].

After appropriate work-up, coordination compounds **7** and **9**–**15** could be isolated as red (**7**, **9**), green (**10**), purple (**12**), or orange (**11**, **13**–**15**) solids, which dissolve, for example, in dichloromethane and tetrahydrofuran, while in non-polar solvents, they are insoluble. Ti(III) and Fe(III) complexes **10** and **11** were obtained as poorly soluble, paramagnetic compounds. Attempts to enhance their solubility by introducing alkyl or alkyloxy chains failed, due to the described difficulties by attaching such groups to the β-diketonato backbone.

The newly prepared complexes are stable towards air, light, and moisture both in the solid state and in solution. They were characterized by elemental analysis, IR and NMR (^1^H, ^13^C{^1^H}, if possible) spectroscopy, and high resolution ESI-TOF mass spectrometry. The spectroscopic and spectrometric data are consistent with their formulation as diferrocenyl β-diketones, β-ketonates, or pyrazoles. In addition, the molecular structures of **5**, **7**, **12**, **13**, **15**, and **16** in the solid state were determined by single crystal X-ray structure analysis. Electrochemical studies (cyclic and square-wave voltammetry) were carried out on **5**–**7** and **9**–**16**. 

The IR spectra are characterized by the appearance of CO vibrations typical for β-diketones (**5**–**7**, **9**) or metal β-diketonates (**10**–**12**) in the range of 1500–1680 cm^−1^ [39,40,102,103]. For the respective diferrocenyl pyrazoles **13**–**15**, representative vibrations could be observed for the N-H (3392 cm^−1^), N-C (ca. 1105 cm^−1^) and N=C units (ca. 1600 cm^−1^) [60].

A distinctive signature of the ^1^H NMR spectra is the appearance of the cyclopentadienyl proton signals between 4.5–5.0 ppm with multiplets or pseudo-triplets for the C_5_H_4_ units with *J_HH_* = 1.9 Hz and a singlet at ca. 4.1 ppm for the C_5_H_5_ protons. The α-hydrogen in β-diketonato complex **12** and pyrazoles **13**–**15** resonates at 6.1–6.6 ppm, while in **7**, it is observed at 4.26 ppm (Experimental). The OH functionality in **5** was observed at 16.4 ppm as a broad signal, due to rapid 1,5-tautomerism, in addition to the CH resonance at 5.9 ppm. The enol form equilibrates with its β-diketo form in a 2:1 ratio. The kinetics of this equilibrium have been studied recently [84].

In the ^13^C{^1^H} NMR spectra, the β-diketonate CO groups give rise to a distinctive resonance signal at ca. 200 ppm. For the ferrocenyl groups, in total, four signals are observed between 65–80 ppm, which is typical for the C_5_H_4_ and C_5_H_5_ perimeters (Section 4) [36,37,38,39,40,41].

In the ESI-TOF, mass spectrometric studies the protonated molecular ion peak [M + H]^+^ is found (Section 4.9). This confirms the formation of **10** as a neutral Ti(III) compound, which requires an additional charge to be detected, instead of an already positively charged Ti(IV) species. As shown within the formation of **16**, containing the respective diphenyl backbone, oxidation towards the Ti(IV) state is common.

### 2.2. Molecular Solid-State Structure

**Figure 1:**
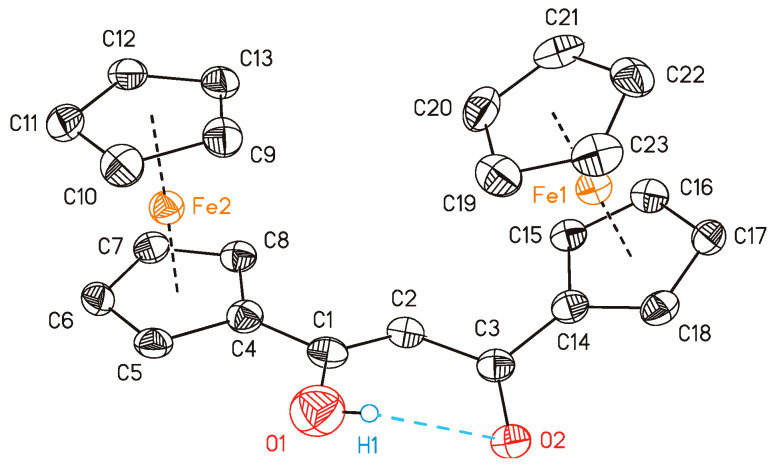
ORTEP (30% probability level) of the molecular structure of **5** with the atom numbering scheme. C-bonded hydrogen atoms have been omitted for clarity. Selected bond distances (Å), angles, and torsion angles (°): C1−O1 1.452(3), C3=O2 1.235(10), C1=C2 1.369(12), C2−C3 1.477(12), C1−C2−C3 124.4(7), O1−C1−C3−O2 10.6(10), O1−C1−C2−C3 2.4(13), C1−C2−C3−O2 31.8(12). Hydrogen bridge bond properties: O1∙∙∙O2 2.975(12) Å, O1−H1∙∙∙O2 119°.

**Figure 2:**
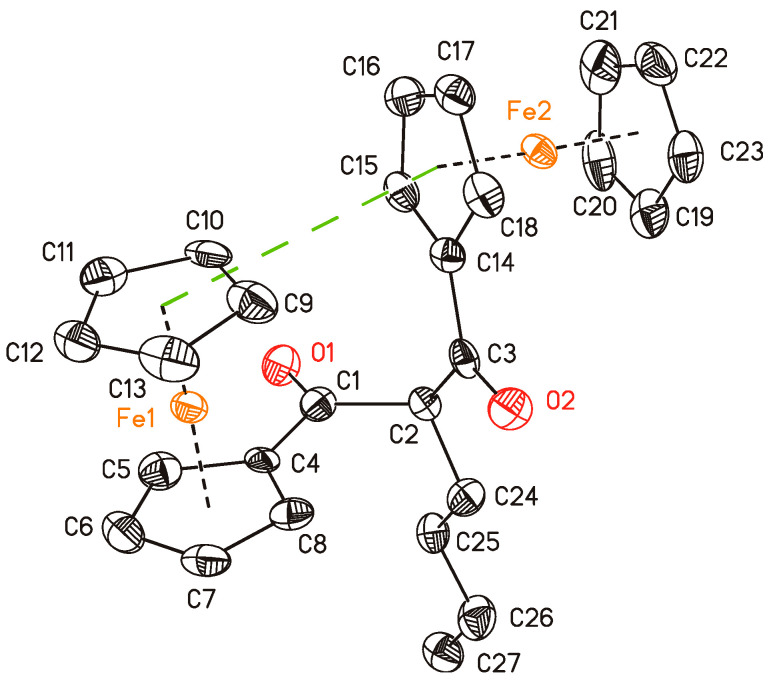
ORTEP (50% probability level) of the molecular structure of **7** with the atom numbering scheme and an intramolecular *T*-shaped CH *π*-interaction (green) between two centroids (Ct). All hydrogen atoms have been omitted for clarity. Selected bond distances (Å), angles, and torsion angles (°): C1=O1 1.222(6), C3=O2 1.224(5), C1−C2 1.480(7), C2−C3 1.457(7), C1−C2−C3 111.6(4), O1−C1−C2−C3 121.1(5), O2−C3−C2−C24 16.1(7); intramolecular *T*-shaped *π*-interaction: Ct∙∙∙Ct 4.520(3) Å; α 75.1(3)° (Ct = centroid of the cyclopentadienyl groups).

**Figure 3:**
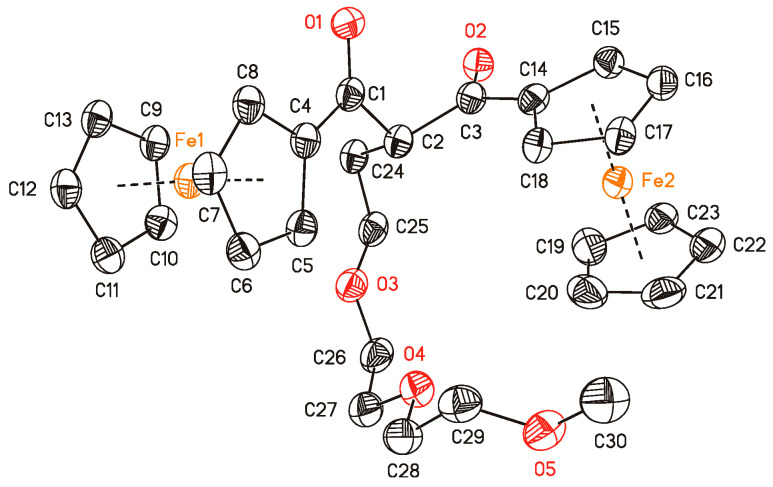
ORTEP (30% probability level) of the molecular structure of **9** with its atom numbering scheme. Hydrogen atoms and a second crystallographically independent molecule of the asymmetric unit have been omitted for clarity. Selected bond distances (Å), angles, and torsion angles (°): C=O 1.197(7)−1.213(8), C1−C2/C2−C3 1.469(9)−1.478(9), C1−C2−C3 108.2(4)/108.4(6), O1−C1−C2−C3 38.9(6)/34.9(7), C1−C2−C3−O2 104.9(6)/103.1(8).

**Figure 4:**
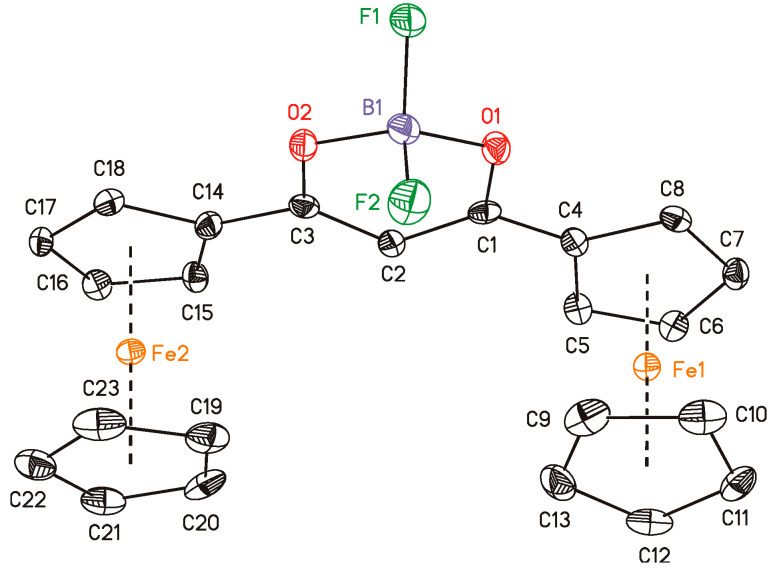
ORTEP (50% probability level) of the molecular structure of **12** with the atom numbering scheme. Hydrogen atoms have been omitted for clarity. Selected bond distances (Å), angles, and torsion angles (°): C1−O1 1.314(3), C3−O2 1.307(3), C1−C2 1.386(3), C2−C3 1.384(3), O−B1 1.488(3)/1.480(3), C1−C2−C3 119.6(2), O1−C1−C3−O2 2.40(18), O1−C1−C2−C3 3.7(4), C1−C2−C3−O2 6.5(4).

**Figure 5:**
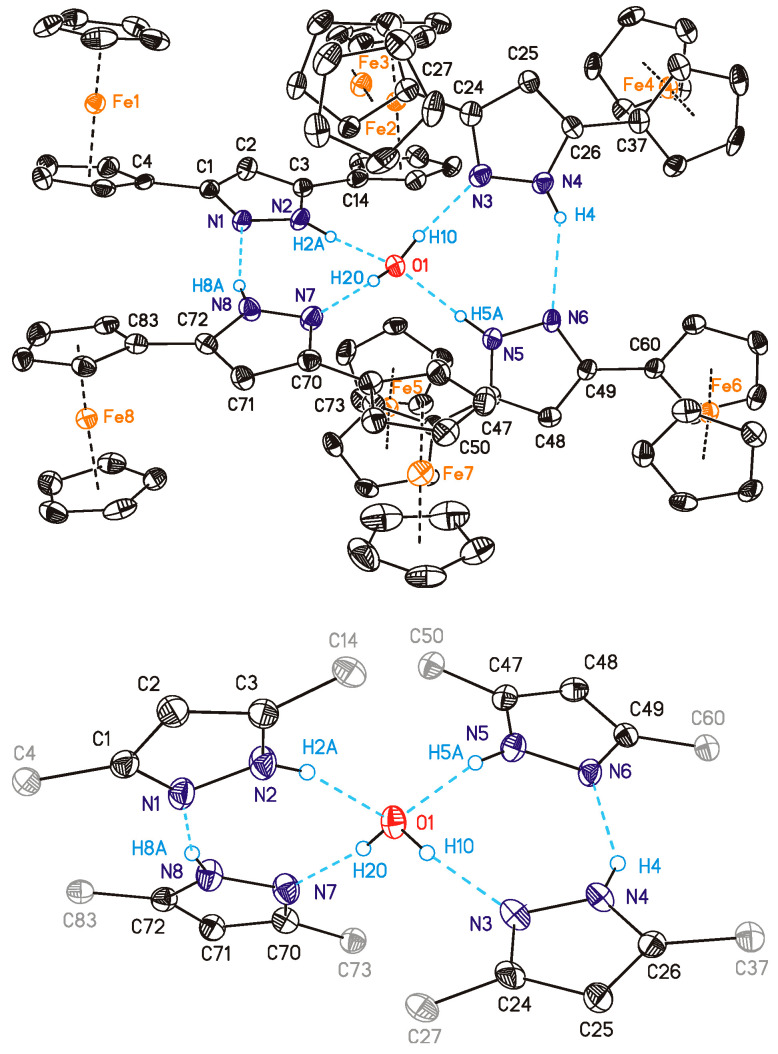
ORTEP (50% probability level) of the molecular structure of **13** (top) with the atom numbering scheme. All C-bonded hydrogen atoms and have been omitted for clarity, as well as the ferrocenyls (pale grey) in the lower graphic. Selected bond distances (Å): C1−N 1.343(4)−1.357(4), C_Py_−C_Py_ 1.375(4)−1.396(4), N−N 1.362(4)−1.367(4).

**Figure 6:**
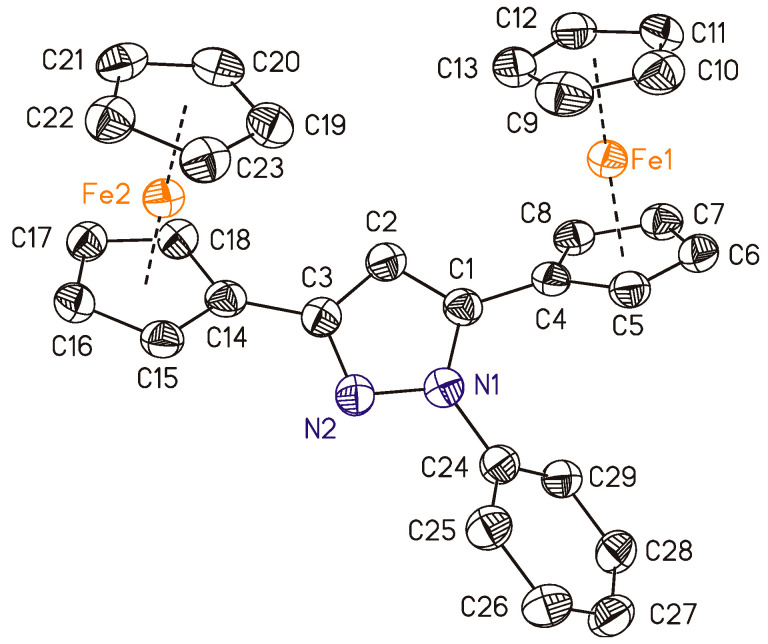
ORTEP (50% probability level) of the molecular structure of **15** with the atom numbering scheme. Hydrogen atoms have been omitted for clarity. Selected bond distances (Å): N1−C1 1.373(4), C1=C2 1.373(4), C2−C3 1.404(4), C3=N2 1.332(4), N1−N2 1.375(3).

**Figure 7:**
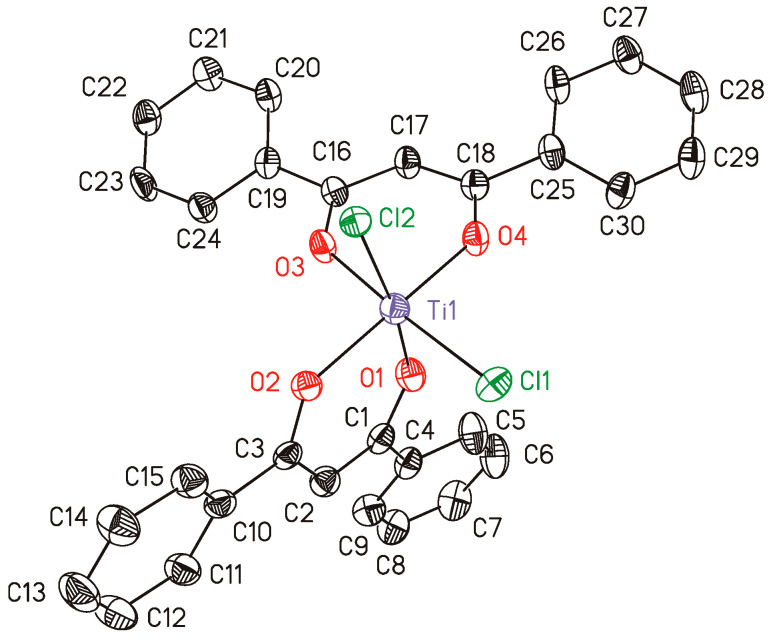
ORTEP (30% probability level) of the molecular structure of **16** with the atom numbering scheme. Hydrogen atoms have been omitted for clarity. Selected bond distances (Å), angles, and torsion angles (°): C−O 1.277(5)−1.291(5), C1−C2/C2−C3 1.379(5)−1.393(6), Ti−O1/O3 1.973(3)/1.970(3), Ti−O2/O4 1.928(3)/1.924(3), Ti−Cl1 2.2867(13), Ti−Cl2 (2.3033(12), C1−C2−C3 123.0(4)/123.6(6), O1−C1−C2−C3 6.6(6)/1.3(6), C1−C2−C3−O2 3.1(7)/0.9(6).

The molecular structures of **5**, **7**, **9**, **12**, **13**, **15,** and **16** in the solid state have been determined by single-crystal X-ray diffraction analysis (Figure 1, Figure 2, Figure 3, Figure 4, Figure 5, Figure 6 and Figure 7). Crystal and structure refinement data, and crystallization conditions are displayed in Section 4. Selected bond lengths (Å), angles (°), and torsion angles (°), as well as plane intersections are listed in Tables S1 and S2 (see the ESI). 

The metal-organic complexes crystallize in triclinic (*P*−1, **13**), monoclinic (*P*2_1_/*c*, **9**, **12**; *P*2_1_/*n*, **7**, **15**, **16**), and non-centrosymmetric orthorhombic (*P*2_1_2_1_2_1_, **5**; abs. struct. param.: 0.000(16) [104]) space groups, with one molecule in the asymmetric unit, except for **9** containing two. Compound **16** was obtained as a methanol solvate, whereby disorder required removal of the packing solvent with the SQUEEZE procedure implemented in the PLATON program package (see Section 4) [105]. The asymmetric unit of **13** is described by a (**13**)_4_·H_2_O arrangement (see below).

In **7**, **9**, **10**, and **12,** identical C−O distances for both CO groups are found, whereby their lengths increase from non-complexed alkyl- (**7**) and alkoxy-substituted (**9**) derivatives (1.197(7)–1.224(5) Å) to BF_2_ (**12**) and Ti (**16**) (1.277(5)–1.314(3) Å). Consequently, the negative charge in the latter two species is delocalized through the β-diketonato system, which also results in equivalent C−C bond lengths (1.379(6)–1.393(6) Å). In contrast, enol **5** shows an alternating sequence of single and double bonds, which also leads to the assignment of O1 as the hydroxy (C1−O1 1.452(3) Å) and O2 as the keto-functionality (C3=O2 1.235(10) Å). A crystal structure of the enol form of **5** has previously been reported [106], showing that both ferrocenyls are in an *anti*-orientation towards each other. Herein, crystallization from a chloroform solution resulted in a *syn*-arrangement. The crystallization of the β-diketo form for α-substituted species **7** and **9** is in accordance to literature, where this behavior is exclusively discussed [107,108]. The keto functionalities can either direct in the same (**9**) or opposite directions (**7**), due to the rotational freedom around the an *sp*^3^-hybridized carbon. For the latter species, this is accompanied with an intramolecular *T*-shaped *π*-interaction between a C_5_H_4_ and a C_5_H_5_ ring (Figure 2). [93,94,95] For the theoretical calculations regarding the properties of parallel-displaced and various types of T-shaped π-interactions, see references [109,110]; for examples involving ferrocenes, see [111,112,113,114].

In contrast to **7** and **9**, compounds **5**, **12**, and **16** adopted a co-planar alignment of the central C_3_O_2_ entity. In case of **5**, a rather weak hydrogen bond restricted the O1−C1⋯C3−O2 torsion angle to 28.6(7)°, which is further reduced to 1.9(3)–4.5(3)° for **12** and **16**, containing stronger O−M bonds (M = Ti, B). The distribution of the negative charge in tetrahedral BF_2_-containing **12** is accompanied by equal B−O (1.488(3) and 1.480(3) Å) and B−F bond lengths (1.488(3) and 1.480(3) Å). In contrast, the *cis*-dichloro substituents in the octahedral coordinated titanium complex **16** caused an elongation of the Ti−O bonds from O−Ti−O (Ti−O: 1.928(3) and 1.924(3) Å) to O−Ti−Cl bonds (Ti−O: 1.973(3) and 1.970(3) Å), due to the stronger trans-influence of the chloride substituent. In contrast, a recently published solid-state structure of **16**, which crystallized in the space group *Pbca* (measured at 193 K) [115], did not show this trans-influence of the chloro substituents, due to a lower C−C bond precision. Nevertheless, a similar trans-influence can be observed for literature-known bis- or tris(β-diketonato) complexes [116,117,118,119]. It should be noted that all bis(β-diketonato)TiX_2_ complexes (X = Cl, O, alkoxy, aryloxy) exclusively crystallize as their cis-derivatives [115]. 

The β-diketonato motif in bora- and titana-cyclic structures **12** and **16**, formed upon coordination towards BF_2_ and TiCl_2_, underwent distortions in order to comply with the requirements for tetrahedral (**12**) and octahedral (**16**) coordination environments, which requires a smaller cavity between both oxygen donors. The comparably long Ti−O bond distances in **16** compensate the unfavorable small O−Ti−O angles of 83.38(12) and 83.96(12)° and keep the Ti atom in the almost planar β-diketonato motif (out-of-plane shift of only 0.216(5) and 0.065(5) Å). A distortion of the C_3_O_2_-planes results in high rms deviations of 0.0244 and 0.107, resulting in apparently higher out-of-plane shifts of the Ti atom. Instead, the by ~0.5 Å shorter B−O bond lengths in **12** squeeze the backbone to achieve the required shortening of the O···O distances by ~0.15 Å from 2.595(4)/2.605(4) (**16**) to 2.450(2) Å in **12**. Although the tetrahedral coordination sphere allows for a larger O−B−O angle of 111.2(2)°, the BF_2_ fragment is shifted out of the planar β-diketonato entity by 0.368(4) Å (vide supra). If expressed by the C2−C−O−B1 torsion angels, a bend of up to 15.7(3)° is observed, which is comparable to other heterocyclic structures where similar out-of-plane shifts prevent involvement of the heteroatom into the π-conjugation [120,121,122].

The ferrocenyl C_5_H_4_- and the adjacent carbonyl functionalities intersect rather co-planar in **5**, **7**, **9,** and **12** with a maximum of 10.6(10)°, whereas the phenyls in **16** and one ferrocenyl group in **5** are slightly rotated out of planarity by up to 23.1(8)°. 

The pyrazolyl entity in **15** intersects with the adjacent ferrocenyls by 45.65(13) and 36.22(11)°, which is slightly larger than for the recently published *N*-benzyl derivative (26.5 and 29.4°) [60]. For the *N*-phenyl (**15**) and *N*-benzyl [60] pyrazoles, single and double bonds within the aromatic heterocycle could be distinguished (Figure 6), contrary to the 1*H*-derivative **13**. Therein, tautomerism causes a similar occupations for hydrogens to be placed at either of the pyrazole′s nitrogen atoms. This affects the whole hydrogen bond network within the (**13**)_4_·H_2_O arrangement, which is established between four molecules of **13** surrounding one central molecule of water in a tetrahedral geometry (Table 1). This cluster is further stabilized by *T*-shaped π-interactions between the Fc groups, which are overlapping each other in the upper graphic in Figure 5 (Figure S1). In order to avoid refinement of the hydrogen bond network over all possible sets of sites, the positioning of the hydrogen atoms followed the highest residual electron density signal (Q-peak) and was extended over the rest of the fragment accordingly. However, the small differences between both possible isomers explains the absence of clear C−C and N−C single and double bonds within the pyrazolyl cores of (**13**)_4_·H_2_O.

In a simplified representation of the hydrogen bridge-bond pattern in Figure 5 and the corresponding geometric properties (Table 1), it can also be seen that two rather co-planar (15.24(5) and 14.89(15)°) and two quite perpendicular plane intersections (68.01(12) and 69.17(13)°) of central heterocyclic cores towards each other are present. The ferrocenyl units between two coplanar moieties are always directed away from the adjacent fragment, whereas a *syn*-fashion for N1- and N7-based building blocks and an *anti*-rotation for N3 and N5 pyrazoles is perceived.

### 2.3. Electrochemistry

**Figure 8:**
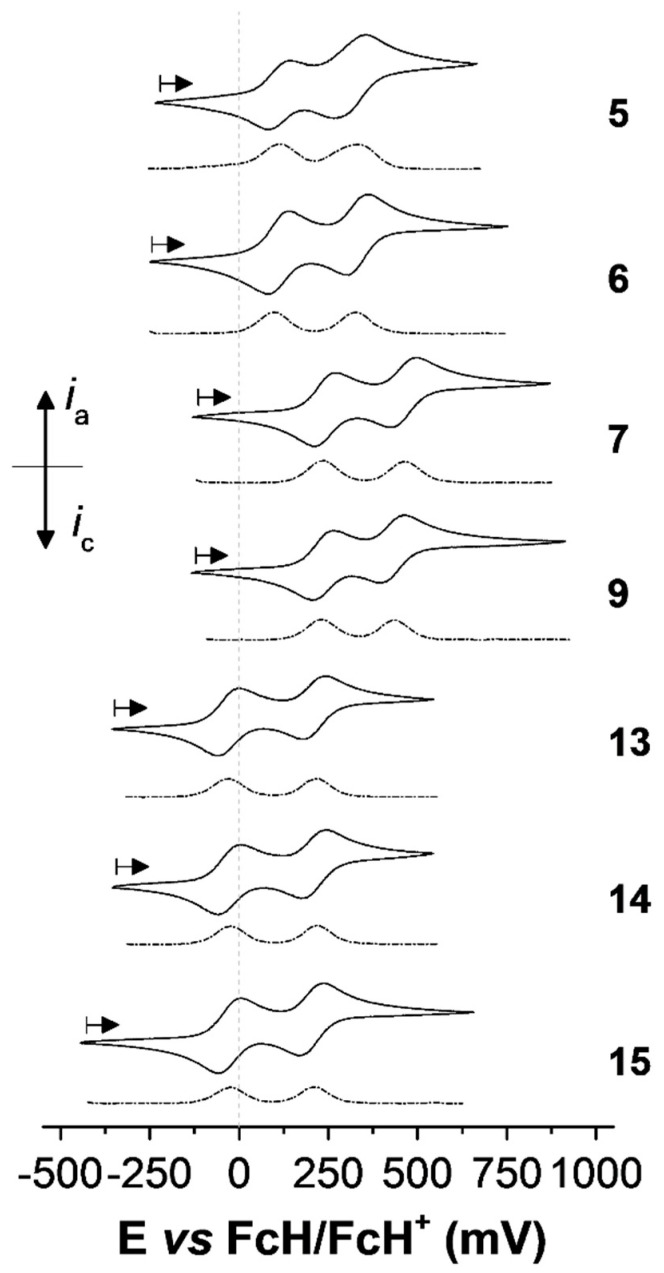
Cyclic voltammograms (solid lines: scan rate 100 mV s^−1^) and square-wave voltammograms (dotted lines: step-height 25 mV, pulse-width 5 s, amplitude 5 mV) of **5**‒**7**, **9**, and **13**‒**15** in dichloromethane solutions (1.0 mmol·L^−1^) at 25 °C measured with a glassy carbon working electrode. Supporting electrolyte 0.1 mol·L^−1^ of [NBu_4_][B(C_6_F_5_)_4_]).

**Figure 9:**
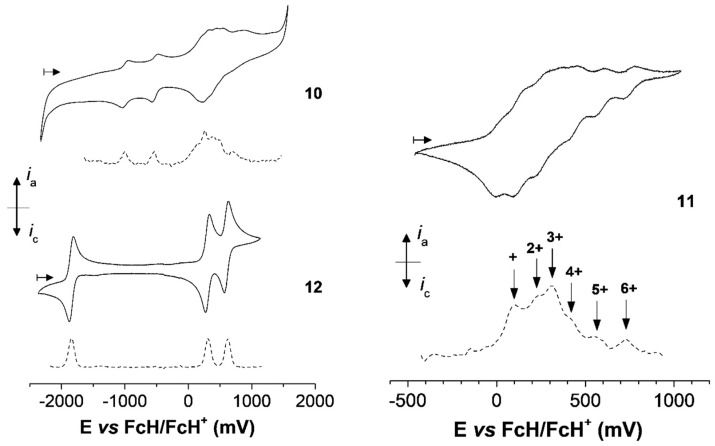
Cyclic voltammograms (solid lines: scan rate 100 mV s^−1^) and square-wave voltammograms (dotted lines: step-height 25 mV, pulse-width 5 s, amplitude 5 mV) of **10** (left, top), **11** (right), and **12** (left, bottom) in dichloromethane solutions (1.0 mmol·L^−1^) at 25 °C measured with a glassy carbon working electrode. Supporting electrolyte 0.1 mol·L^−1^ of [NBu_4_][B(C_6_F_5_)_4_]).

The redox behavior of **5**‒**7** and **9**‒**16** has been determined by cyclic voltammetry (=CV) and square-wave voltammetry (=SWV) (Figure 8 and Figure 9). The electrochemical measurements were carried out in anhydrous dichloromethane solutions containing [NBu_4_][B(C_6_F_5_)_4_] (0.1 mol∙L^−1^) as a supporting electrolyte under inert conditions at 25 °C (Section 4.4) [123,124]. In contrast to smaller counter ions including [PF_6_]^−^ or [Cl]^−^, the [B(C_6_F_5_)_4_]^−^ anion stabilizes highly charged species in solution, minimizing ion pairing effects. The shielding of the electrostatic interactions between the redox-active groups is realized by ion pairing with the electrolyte’s counter-ion. Hence, minimization of this effect leads to an increase of the observed redox potentially splitting [68,125,126].

All potentials are referenced to the FcH/FcH^+^ (FcH = Fe(η^5^-C_5_H_5_)_2_) redox couple [127]. The CV data at a scan rate of 100 mV s^−1^ are summarized in Table 2. 

Compounds **5**–**7**, **9,** and **13**‒**15** show two reversible one-electron redox processes, confirming separate oxidation of the Fc groups. The diferrocenyl β-diketone **5** was first electrochemically studied in 1999 using [NBu_4_][PF_6_] as an electrolyte (*E*_1_°′ = 188 mV and *E*_2_°′ = 297 mV) in order to obtain group electro-negativities by means of cyclic voltammetry [126,128,129,130]. For better comparison with our data, we repeated the CV measurements in the presence of the weak coordinating electrolyte [NBu_4_][B(C_6_F_5_)_4_] [68,125,126]. As it can be seen from Figure 8, compound **5** shows its second redox event to be broadened, most likely due to keto-enol tautomerism in the mixed-valent state [37,38,41]. This differs from **6**, since this compound only features one β keto-group. The butyl or glycol groups in α-position of the β-diketones **7** and **9** result in a shift of the first redox potential to more positive values (**7**, *E*_1_°′ = 240 mV; **9**, *E*_1_°′ = 235 mV) in comparison to **5** (*E*_1_°′ = 110 mV). This leads to the assumption that the Fc nearby the enol group possesses a higher electron density, and thus, most likely it will be oxidized first. As it can be seen from Table 2, the redox separations of all analyzed β-diketones are with Δ*E*°′ ca. 200 mV similar (Table 2). Most of these redox-separations, however, are likely to be caused by electrostatic repulsion of the ferrocenium entities in close spatial proximity. In addition, the redox separation for **6** and **13**–**15** is slightly higher, since the two Fc units are not in chemically equivalent positions. The redox behavior of the 3,5-diferrocenyl-functionalized pyrazoles **13**‒**15** is very similar to each other (*E*_1_°′ = 30 mV, Δ*E*°′ = ca. 210 mV; Table 2), proving negligible influence of the N-bonded hydrogen (**13**) methyl (**14**) or phenyl (**15**) group on the charge transfer process or the electrostatics in the mono-oxidized mixed-valent compounds [**13**‒**15**]^+^.

β-Diketone **5** was used as ligand in the synthesis of titanium, iron, and boron β-diketonates **10**–**12** (Scheme 2) in order to study their electrochemical behavior and, hence, the influence of the metal ion on the charge transfer between the ferrocene/ferrocenium groups in the mixed-valent species.

Complex **12** exhibits three reversible one-electron processes. While at a potential of 300 and 600 mV the two Fc/Fc^+^ processes are observed, the reversible redox process at −1845 mV (Δ*E*_p_ = 64 mV) represents a one-electron reduction of the *π*-system of the six membered C_3_O_2_B cycle. The delocalized character of these *π* electrons in addition lead to a much better electronic coupling of the ferrocenyl units, and hence, the redox separation is increased to 300 mV, when compared to the parent diketonate **5** (Δ*E*°′ = 200 mV).

As a result of the poor solubility of ferrocenyl-functionalized metal β-diketonates in, for example, dichloromethane or acetonitrile, only a few examples of this family of compounds have been characterized electrochemically in the literature, including [Cu(κ^2^*O*,*O*′-FcC(O)CHC(O)Fc)_2_] [40] and [Al(κ^2^*O*,*O*′-FcC(O)CHC(O)Fc)_3_] [41], respectively. 

Complex **10** exhibits two titanium-related reversible redox processes at *E*_1_°′ = −985 mV and *E*_2_°′ = −520 mV, respectively. Comparison with **16** (Figure S2, see the Supporting Information) confirms that the wave at −520 mV can be assigned to the reversible oxidation of Ti^III^ to Ti^IV^ (Δ*E*_p_ = 64 mV), while the redox event at −985 mV (Δ*E*_p_ = 60 mV) corresponds to the reduction of Ti^III^ to Ti^II^ (Figure 9), which is in agreement with the one found for [Ti_2_L_3_] (L = 1,3-bis(3-phenyl-3-oxopropanoyl)benzene) at ca. −1100 mV [131]. However, for **16** no reduction process could be observed in the appropriate measured frame (1000 to ‒1500 mV). In addition to the Ti-related processes in **10**, the six ferrocenyl-related redox events are poorly resolved occurring in the potential range between 125 and 670 mV, due to the low solubility of the complex. Square-wave voltammetry allows us to assign five individual processes at 140 mV, 260 mV (2 e^−^), 385 mV, 485 mV, and 670 mV (Figure 9).

In contrast, the ferrocenyl-related oxidation processes for iron(III) complex **11** are much better resolved, and hence, the SWV of **11** allows us to identify the respective formal potentials at 100, 230, 305, 430, 560, and 730 mV (Figure 9). For the analog [Al(κ^2^*O*,*O*′-FcC(O)CHC(O)Fc)_3_] complex, a comparable redox behavior was observed under similar measurement conditions (*E*_1_°′ = 33, *E*_2_°′ = 123, *E*_3_°′ = 304, *E*_4_°′ = 432, *E*_5_°′ = 583, *E*_6_°′ = 741 mV) [41]. In comparison to [Al(FcC(O)CHC(O)Fc)_3_], the first oxidation in **11** takes place at a higher potential (*E*_1_°′ = 100), due to lower electron density at the Fc′s. Reduction processes for Fe^III^ in **11** could, however, not be found up to −2200 mV (Figure S3, see the ESI).

In order to get a deeper insight into the spectroscopic details of the mixed-valent species [**5**]^+^, [**6**]^+^ and [**12**–**15**]^+^ in situ UV-Vis/NIR, spectro-electrochemical measurements have been carried out. However, the low solubility of complexes **10** and **11** did not allow for spectro-electrochemical measurements to be carried out. The spectro-electrochemical studies were performed by stepwise increase of the potential from −400 to 1200 mV (step heights: 25, 50 or 100 mV) vs. Ag/AgCl in an optically transparent thin layer electrochemistry cell (=OTTLE) [132]. Dichloromethane solutions of **5**, **6,** and **12**–**15** (0.02 M) containing [NBu_4_][B(C_6_F_5_)_4_] (0.1 M) as the supporting electrolyte were used [123,124]. Thereby, the stepwise generation of mixed-valent [**5**]^+^, [**6**]^+^, and [**12**–**15**]^+^ and homo-valent [**5**]^2+^, [**6**]^2+^, and [**12**‒**15**]^2+^ occurred. The spectra of [**5**]^+^ and [**6**]^+^ are shown in the Supporting Information (Figures S4 and S5, see the ESI).

**Figure 10:**
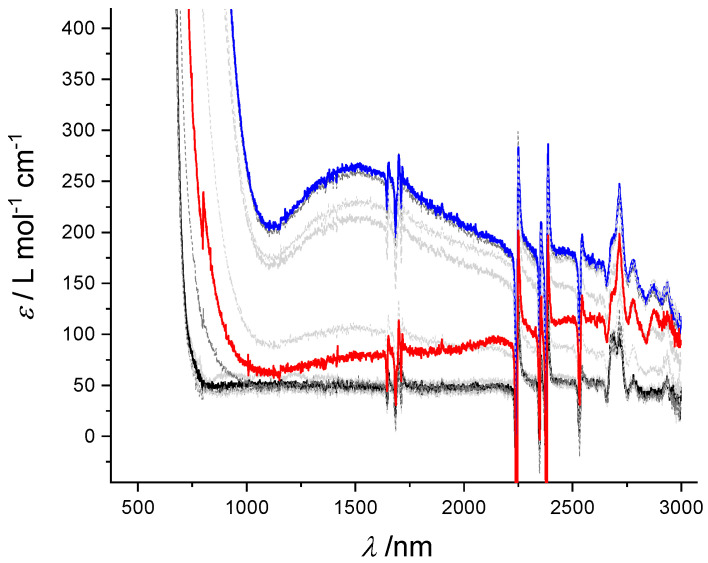
UV-Vis/NIR spectra of **12** in a dichloromethane solution (2.0 mmol·L^−1^) at rising potentials (−200 mV to 1200 mV vs. Ag/AgCl) at 25 °C; supporting electrolyte 0.1 mol·L^−1^ of [NBu_4_][B(C_6_F_5_)_4_] black −200 mV (**12**); blue 750 mV ([**12**]^+^); red 1200 mV ([**12**]^2+^).

During the oxidation of **5** and **6** an IVCT absorption of negligible strength can be seen. The extinction coefficient of this band, however, is lower than 50 L·mol^−1^·cm^−1^, therefore the electronic coupling between the Fc/Fc^+^ is very weak (Figures S4 and S5). The introduction of the BF_2_ unit in **12** led to an increase in the extinction of the IVCT band (*ν_max_* = 6250 cm^−1^, *Δν_½_* = 4725 cm^−1^, *ε_max_* = 240 L·mol^−1^·cm^−1^) (Figure 10, Figure S6), corresponding to a weakly coupled class II system according to the classification of Robin and Day [133]. The formation of the six-membered C_3_O_2_B ring introduced some rigidity in the π-bridge, and hence, the electronic coupling of the ferrocenyl termini became stronger. 

For pyrazoles **13**–**15** no IVCT band of considerable strength could be found in any oxidation state, demonstrating that the 240 mV redox separation is mainly caused by electrostatic interactions.

## 3. Conclusions

The synthesis and characterization of diferrocenyl-substituted β-diketones, 1*R*-pyrazoles, and β-diketonato metal complexes of general type FcC(O)C(R)C(OH)Fc (Fc = Fe(η^5^-C_5_H_4_)(η^5^-C_5_H_5_); R = H, **5**; *^n^*Bu, **7**; CH_2_CH_2_(OCH_2_CH_2_)_2_OMe, **9**), 1-R-3,5-Fc_2_-*^c^*C_3_HN_2_ (R = H, **13**; Me, **14**; Ph, **15**), [M(κ^2^*O*,*O*′-FcC(O)CHC(O)Fc)_n_] (M = Ti, *n* = 3, **10**; M = Fe, *n* = 3, **11**; M = BF_2_, *n* = 1, **12**), and [TiCl_2_(κ^2^*O*,*O*′-PhC(O)CHC(O)P)_2_] (**16**) is discussed. The molecular solid-state structures of **5**, **7**, **9**, **12**, **13**, **15,** and **16** were determined by single-crystal X-ray diffraction studies, verifying the predicted structures. Alkyl substitution of the β-diketone led to an increased electron density at C_α_ and, hence, resulted in the formation of the diketo form in solution and solid state, in contrast to the H-substituted derivative **5,** where the enol form was moreover present, resulting in an intramolecular hydrogen bond in the solid state. In diferrocenyl 1*Ph*-pyrazole **15**, a differentiation between single and double bonds in the heterocyclic core could be observed. In contrast, the NH-derivative **13** crystallized as a tetrameric structure surrounding one central molecule of water, where all five molecules were connected via hydrogen bonds.

Electrochemical measurements confirmed that the ferrocenyls in **5**–**7**, **9,** and **12**–**15** could be oxidized separately; however, the redox separation of 200–250 mV is mainly caused by electrostatic interactions. In addition, for complexes **6** and **13**–**15**, the ferrocenyl units are in chemically non-equivalent positions, and hence, the observed redox separation is increased. The Ti and Fe complexes **10** and **11** showed a convoluted redox behavior, since the six ferrocenyl units are oxidized in a close potential range; however, square-wave voltammetry allowed us to estimate the formal potential of each ferrocenyl oxidation process. In addition, complex **10** showed two titanium-centered redox processes at −985 and −520 mV, corresponding to Ti^II^/Ti^III^ and Ti^III^/Ti^IV^ redox couples, respectively. 

Compound **12** showed an increased redox separation between the Fc units upon introduction of BF_2_, due to a more rigid backbone, which allows for a better conjugation through the C_3_-π-bridge. Spectro-electrochemical UV/Vis-NIR measurements confirmed a stronger electronic coupling in mixed-valent [**12**]^+^ between Fc/Fc^+^ than for non-coordinated diketone [**5**]^+^.

## 4. Materials and Methods

### 4.1. General Procedures

All reactions were carried out under an atmosphere of argon using standard Schlenk techniques. Tetrahydrofuran was purified by distillation from sodium/benzophenone ketyl. Hexane was purified with a MBRAUN SBS-800 purification system. Dichloromethane was purified by distillation from CaH_2_. For column chromatography, alumina with a particle size of 90 μm (standard, Merck KgaA) or silica with a particle size of 40–60 μm (230–400 mesh (ASTM), Fa. Macherey-Nagel) was used. As filtration support Zeolithe (Riedel de Häen) was applied.

### 4.2. Instruments

Infrared spectra were recorded at ambient conditions with a FT-Nicolet IR 200 equipment or as ATR-FTIR spectra by using a Biorad FTS-165 or a Nicolet iS 10 spectrometer from Thermo Scientific. NMR spectra (500.3 MHz for ^1^H, 125.7 MHz for ^13^C, 160.5 MHz for ^11^B) were recorded using a Bruker Avance III 500 FT-NMR spectrometer at ambient temperature. Chemical shifts are reported in ppm downfield from tetramethylsilane with the solvent as reference signal (^1^H NMR: δ (CDCl_3_) = 7.26 ppm; ^13^C{^1^H} NMR: δ (CDCl_3_) = 77.16 ppm). The melting points were determined with a Gallenkamp MFB 595 010 M melting point apparatus. Elemental analyses were performed with a Thermo FlashEA 1112 Series instrument (ThermoFisher). High-resolution mass spectra were recorded using a micrOTOF QII Bruker Daltonite workstation. 

### 4.3. Crystallography 

Data were collected with an Oxford Gemini S diffractometer at ≤120 K using Mo K_α_ (λ = 0.71073 Å) radiation. The structures were solved by direct methods and refined by full-matrix least square procedures on *F*^2^ with SHELXL-2013 [134,135]. All non-hydrogen atoms were refined anisotropically, and a riding model was employed in the treatment of the hydrogen atom positions. Graphics of the molecular structures have been created using ORTEP [136].

The acidic hydrogen atom in **5** has been refined as an idealized OH group with the torsion angle derived from electron density (AIFX 147). In **13**, idealized aromatic hydrogens (AFIX 43) were used for the calculation of the N−H functionalities, whereas the positions of the water hydrogens were derived from residual density and fixed by using DFIX and DANG instructions. The titanium(III) complex **16** was crystallized from methanol and contained disordered solvent molecules in the asymmetric unit. However, attempts to refine them over several sets of sites have not been successful, and thus, they have been omitted by applying the SQUEEZE [105] procedure of the PLATON [137,138] program package. Solvent-accessible voids of 760 Å^3^ per unit cell were found, and 113 electrons have been omitted, which corresponds to slightly less than two molecules of methanol within the asymmetric unit of **16**.

Crystal data for **5**: C_23_H_20_Fe_2_O_2_, *M* = 440.09 g mol^−1^, orthorhombic, *P*2_1_2_1_2_1_, *λ* = 0.71073 Å, *a* = 8.7957(8) Å, *b* = 9.6795(9) Å, *c* = 21.569(3) Å, *V* = 1836.3(3) Å^3^, *Z* = 4, *ρ*_calcd_ = 1.592 Mg m^−3^, *μ* = 1.595 mm^−1^, *T* = 120.00(10) K, *θ* range 3.530–24.990°, 5616 reflections collected, 3005 independent reflections (*R*_int_ = 0.0330), *R*1 = 0.0614, *wR*2 = 0.1496 (*I* > *2σ*(*I*)), absolute structure parameter 0.000(16).

Crystal data for **16**: C_30_H_22_Cl_2_O_4_Ti, *M* = 565.27 g mol^−1^, monoclinic, *P*2_1_/*n*, *λ* = 0.71073 Å, *a* = 14.3372(7) Å, *b* =15.3425(8) Å, *c* = 14.6145(7) Å, *β* = 97.054(5)°, *V* = 3190.4(3) Å^3^, *Z* = 4, *ρ*_calcd_ = 1.177 Mg m^−3^, *μ* = 0.464 mm^−1^, *T* = 120.00(14) K, *θ* range 3.253–24.999°, 13796 reflections collected, 5554 independent reflections (*R*_int_ = 0.0541), *R*1 = 0.0651, *wR*2 = 0.1556 (*I* > *2σ*(*I*)).

### 4.4. Electrochemistry 

Electrochemical measurements on 1.0 mmol·L^−1^ solutions of the analytes in anhydrous, air free dichloromethane containing 0.1 mol∙L^−1^ of [NBu_4_][B(C_6_F_5_)_4_] as a supporting electrolyte were conducted under a blanket of purified argon at 25 °C utilizing a Radiometer Voltalab PGZ 100 electrochemical workstation combined with a personal computer [128,139,140]. A three-electrode cell, which utilized a Pt auxiliary electrode, a glassy carbon working electrode (surface area 0.031 cm^2^), and an Ag/Ag^+^ (0.01 mol∙L^−1^ AgNO_3_) reference electrode mounted on a Luggin capillary were used. The working electrode was pretreated by polishing on a Buehler microcloth first with a 1 μm and then with a 1/4 μm diamond paste. The reference electrode was constructed from a silver wire inserted into a solution of 0.01 mol∙L^−1^ [AgNO_3_] and 0.1 mol∙L^−1^ [NBu_4_][B(C_6_F_5_)_4_] in acetonitrile in a Luggin capillary with a CoralPor tip. This Luggin capillary was inserted into a second Luggin capillary with a CoralPor tip filled with a 0.1 mol∙L^−1^ [NBu_4_][B(C_6_F_5_)_4_] solution in dichloromethane. Successive experiments under the same experimental conditions showed that all formal reduction and oxidation potentials were reproducible within ±5 mV. Experimentally, potentials were referenced against an Ag/Ag^+^ reference electrode, but results are presented referenced against ferrocene as an internal standard as required by IUPAC [125,126]. When decamethylferrocene was used as an internal standard, the experimentally measured potentials were converted into *E* vs. FcH/FcH^+^ by addition of −614 mV [141,142]. Data were then manipulated on a Microsoft Excel worksheet to set the formal reduction potentials of the FcH/FcH^+^ couple to Δ*E*°′ = 0.0 V. Ferrocene itself showed a redox potential of 220 mV vs. Ag/Ag^+^ (Δ*E*_p_ = 61 mV) within the measurements [143,144]. The cyclic voltammograms were taken after typical three scans and are considered to be steady-state cyclic voltammograms in which the signal pattern differs not from the initial sweep.

UV/Vis-NIR measurements were carried out in an OTTLE (=optically thin-layer electrochemistry) cell with quartz windows similar to that described previously [132] in anhydrous dichloromethane solutions containing 2.0 mmol∙L^−1^ analyte and 0.1 mol∙L^−1^ of [NBu_4_][B(C_6_F_5_)_4_] as a supporting electrolyte using a Varian Cary 5000 spectrophotometer at 25 °C. The working electrode Pt-mesh, the AgCl-coated Ag wire for reference, and the Pt-mesh auxiliary electrode are melt-sealed into a polyethylene spacer. The values obtained by deconvolution could be reproduced within *ε*_max_ = 100 L·mol^−1^·cm^−1^, *ν*_max_ = 50 cm^−1^, and Δ*ν*_1/2_ = 50 cm^−1^. Between the spectroscopic measurements, the applied potentials have been increased step-wisely using step heights of 25, 50, or 100 mV. At the end of the measurements, the analyte was reduced at −400 mV for 30 min, and an additional spectrum was recorded to prove the reversibility of the oxidations.

### 4.5. Reagents

[NBu_4_][B(C_6_F_5_)_4_] was prepared by metathesis of lithium tetrakis(pentafluorophenyl)borate etherate (Boulder Scientific) with tetra-*n*-butylammonium bromide according to reference [129]. All other chemicals were purchased from commercial suppliers and were used without further purification. Ethyl ferrocenecarboxylate **1** was synthesized by mono lithiation of ferrocene with *^t^*BuLi and subsequent addition of ethyl chloroformate [79]. Acetylferrocene (**2**) was synthesized by acylation of ferrocene with acetic anhydride and boron trifluoride etherate [80]. Ferrocenyl ketone **3** was formed by a Friedel-Crafts acylation of ferrocene with hexanoyl chloride and anhydrous aluminum chloride as a catalyst [81]. Hexanoyl chloride used for the synthesis of **3** as well as 2-[2-(2-methoxyethoxy)ethoxy]acetyl chloride, applied in the preparation of **4**, were synthesized by refluxing the appropriate carboxylic acids in thionyl chloride for 6 h and subsequent distillation [82,83]. The Claisen condensation of ferrocenyl ester **1** and ketone **2** to give diketone **5** (and as side product **6**) were performed similar (ethyl ferrocenoate instead of methyl ferrocenoate) to a literature-known procedure [84]. The analytical data of **5** agree well with the ones in references [36,84]. The compound 1,3-diferrocenylpropane-1,3-dionato lithium(I) was obtained as an intermediate in the synthesis of **5**, when the reaction solution was filtered off before quenching with aqueous hydrochloric acid. The compound 1-iodo-2-[2-(2-methoxyethoxy)ethoxy]ethane was synthesized by refluxing 1-bromo-2-[2-(2-methoxyethoxy)ethoxy]ethane in the presence of sodium iodide in acetone for 48 h and subsequent distillation at 160 °C (37 mbar) [86]. The compound 1-bromo-2-[2-(2-methoxyethoxy)ethoxy]ethane was prepared by an Appel reaction of 2-[2-(2-methoxyethoxy)ethoxy]ethanol, tetrabromomethane, and triphenylphosphane [88].

### 4.6. Synthesis of 1-Ferrocenyl-2-[2-(2-methoxyethoxy)ethoxy]ethanone *(**4**)*

Ferrocene (6.62 g, 35.6 mmol) and AlCl_3_ (4.75 g, 35.6 mmol) were dissolved in 70 mL of anhydrous dichloromethane and the thus-obtained reaction solution was then cooled to 0 °C. The compound 2-[2-(2-methoxyethoxy)ethoxy]acetyl chloride (7.0 g, 35.6 mmol) in 20 mL of dichloromethane was added dropwise via a dropping funnel within 30 min. The reaction solution was warmed up to ambient temperature for 2 h and then added in a single portion to an aqueous NaHCO_3_ solution. The organic phase was separated. The aqueous solution was extracted twice with 40 mL (each) of dichloromethane. The organic phases were combined, dried over MgSO_4_, and all volatiles were removed in vacuum. The crude product was purified by column chromatography (column size: 20 × 3 cm, alumina) using a hexane–dichloromethane mixture of ratio 1:1 (*v*/*v*) as eluent. The 4th fraction contained the title compound **4**, which after removal of all volatiles gave a brownish oily liquid. Yield: 11.3 g (32.6 mmol, 92% based on ferrocene).

Anal. Calcd. for C_17_H_22_FeO_4_ (M = 346.20 g∙mol^−1^): C 58.98, H 6.41; found: C 58.91, H 6.57. ATIR (ATR; ṽ in cm^−1^): 3095 (m, ν*_C–H_*), 2875 (m), 1721 (w), 1680 (s), 1556 (m), 1455 (m), 1378 (m), 1361 (m), 1253 (m), 1198 (w), 1105 (s), 1064 (s), 1027 (s), 823 (s, π*_C–H_*, C_5_H_5_), 775 (w). ^1^H NMR (CDCl_3_, *δ* in ppm): 3.38 (s, 3H, CH_3_), 3.56 (m, 2H, CH_2_), 3.67 (m, 2H, CH_2_), 3.73 (m, 2H, CH_2_), 3.78 (m, 2H, C(O)CH_2_), 4.21 (s, 5H, C_5_H_5_), 4.51 (pt, ^3^*J*_H,H_ = 1.9 Hz, 2H, C_5_H_4_), 4.56 (s, 2H, CH_2_), 4.83 (pt, ^3^*J*_H,H_ = 1.9 Hz, 2H, C_5_H_4_). ^13^C{^1^H} NMR (CDCl_3_, *δ* in ppm): 59.2 (CH_3_), 69.2 (C_5_H_4_), 70.1 (C_5_H_5_), 70.7 (CH_2_), 71.0 (CH_2_), 71.1 (CH_2_), 72.1 (CH_2_), 72.5 (C_5_H_4_), 74.6 (CH_2_), 76.3 (*^i^*C-C_5_H_4_), 201 (C=O). HRMS (ESI-TOF, *m*/*z*): calcd. for C_17_H_22_FeO_4_ + H: 347.0940; found 347.0954 [M + H]^+^.

### 4.7. Synthesis of 2-Butyl-1,3-diferrocenyl-1,3-propandione *(**7**)*

Ferrocenyl ketone **3** (1.72 g, 6.05 mmol) was dissolved in 20 mL of anhydrous tetrahydrofuran, and this solution was treated with 3.0 mL (2 M in tetrahydrofuran) of lithium diisopropylamide at 0 °C. The reaction solution was stirred for 2 h at 50 °C and a solution of ethyl ferrocenoate **1** (1.72 g, 6.05 mmol) in 10 mL of tetrahydrofuran was added via a syringe. After 10 h of stirring, the reaction solution was shaken with hydrochloric acid and extracted four times with 60 mL (each) of diethyl ether. The combined organic phases were dried over MgSO_4_, and all volatiles were removed. The crude product was purified by column chromatography (column size: 20 × 3 cm, silica) using a hexane–dichloromethane mixture of ratio 2:1 (*v*/*v*) as eluent. The 2nd fraction contained **7**, which, after removal of all volatiles, gave **7** as a red solid. Yield: 362 mg (0.73 mmol, 40% based on **3**).

Anal. Calcd. for C_27_H_28_Fe_2_O_2_ (M = 496.20 g∙mol^−1^): C 65.35, H 5.69; found: C 65.42, H 5.97. Mp.: 142 °C. IR (KBr, ṽ in cm^−1^): 3094 (m, ν*_C–H_*), 2950 (m), 2923 (m), 2870 (m), 1671 (m), 1642 (s, ν*_C=O_*), 1441 (m),1411 (w), 1374 (m), 1285 (m), 1257 (m), 1107 (m), 1049 (m), 1027 (m), 1003 (m), 852 (m), 827 (m, π*_C–H_*, C_5_H_5_), 734 (m), 545 (m), 521 (m), 508 (m, C_5_H_4_ ring tilt). ^1^H NMR (CDCl_3_, *δ* in ppm): 0.93 (t, ^3^*J*_H,H_ = 7.0 Hz, 3H, CH_3_), 1.38 (m, 4H, CH_2_CH_2_), 2.13 (m, 2H, CH_2_), 4.08 (s, 10H, C_5_H_5_), 4.26 (t, ^3^*J*_H,H_ = 7.2 Hz, 1H, CH), 4.51 (ptd, *J*_H,H_ = 2.6, 1.4 Hz, 2H, C_5_H_4_), 4.54 (ptd, *J*_H,H_ = 2.5, 1.3 Hz, 2H, C_5_H_4_), 4.93 (dpt, *J*_H,H_ = 2.5, 1.3 Hz, 2H, C_5_H_4_), 4.94 (dpt, *J*_H,H_ = 2.6, 1.3 Hz, 2H, C_5_H_4_). ^13^C{^1^H} NMR (CDCl_3_, *δ* in ppm): 14.1 (CH_3_), 22.8 (CH_2_), 30.1 (CH_2_), 30.8 (CH_2_), 65.2 (CH), 70.0 (C_5_H_4_), 70.2 (C_5_H_5_), 70.5 (C_5_H_4_), 72.6 (C_5_H_4_), 72.7 (C_5_H_4_), 79.0 (*^i^C*-C_5_H_4_), 199.9 (C=O). HRMS (ESI-TOF, *m*/*z*): calcd. for C_27_H_28_Fe_2_O_2_ + H: 497.0788; found 497.0862 [M + H]^+^.

Crystal data for **7**: C_27_H_28_Fe_2_O_2_, *M* = 496.19 g mol^−1^, monoclinic, *P*2_1_/*c*, *λ* = 0.71073 Å, *a* = 16.2997(15) Å, *b* = 10.7155(7) Å, *c* = 12.6538(10) Å, *β* = 93.817(8)°, *V* = 2205.2(3) Å^3^, *Z* = 4, *ρ*_calcd_ = 1.495 Mg m^−3^, *μ* = 1.337 mm^−1^, *T* = 119.95(10) K, *θ* range 3.458–24.999°, 9367 reflections collected, 3849 independent reflections (*R*_int_ = 0.1002), *R*1 = 0.0601, *wR*2 = 0.0763 (*I* > *2σ*(*I*)).

### 4.8. Synthesis of 2-(2-[2-(2-Methoxyethoxy)ethoxy]ethyl)-1,3-diferrocenyl-1,3-propandione *(**9**)*

Diferrocenyl diketone **5** (360 mg, 0.82 mmol), 1-iodo-2-[2-(2-methoxyethoxy)ethoxy]ethane (225 mg, 0.82 mmol) and KO*^t^*Bu (92 mg, 0.82 mmol) were dissolved in 20 mL of dimethyl sulfoxide and the thus-obtained reaction solution was stirred for 14 h at 50 °C. Afterwards, it was poured into 100 mL of water and extracted five times with 50 mL (each) of diethyl ether. The combined organic phases were dried over MgSO_4_, and all volatiles were removed in vacuum. The crude product was purified by column chromatography (column size: 20 × 3 cm, silica) using a hexane–dichloromethane mixture of ratio 1:1 (*v*/*v*) as eluent. The 2nd fraction contained **9**, which, after removal of all volatiles, gave **9**. Crystallization from a hexane/dichloromethane mixture of ratio 1:20 (*v*/*v*) at −20 °C afforded **9** as red crystals. Yield: 310 mg (0.53 mmol, 64% based on **5**).

Anal. Calcd. for C_30_H_34_Fe_2_O_5_ (M = 586.28 g∙mol^−1^): C 61.46, H 5.85; found: C 61.25, H 6.04. Mp.: 74 °C. IR (KBr, in cm^−1^): 3098 (m, ν*_C–H_*), 3089 (m, ν*_C–H_*), 2884 (m), 1677 (s, ν*_C=O_*), 1656 (s, ν*_C=O_*), 1449 (s), 1410 (m), 1374 (s), 1336 (m), 1295 (m), 1265 (m), 1235 (m), 1132 (m), 1107 (s), 1047 (m), 1026 (m), 991 (m), 898 (m), 833 (s, π*_C–H_*, C_5_H_5_), 658 (m), 532 (m, C_5_H_4_). ^1^H NMR (CDCl_3_, *δ* in ppm): 2.42 (q, ^3^*J*_H,H_ = 6.1 Hz, 2H, CH_2_), 3.37 (s, 3H, CH_3_), 3.55 (m, 4H, CH_2_), 3.65 (m, 6H, CH_2_), 4.11 (s, 10H, C_5_H_5_), 4.50 (ptd, *J*_H,H_ = 2.6, 1.4 Hz, 2H, C_5_H_4_), 4.53 (ptd, *J*_H,H_ = 2.5, 1.3 Hz, 2H, C_5_H_4_), 4.57 (t, ^3^*J*_H,H_ = 6.9 Hz, 1H, CH), 4.92 (dpt, *J*_H,H_ = 2.5, 1.2 Hz, 2H, C_5_H_4_), 4.94 (dpt, *J*_H,H_ = 2.6, 1.3 Hz, 2H, C_5_H_4_). ^13^C{^1^H} NMR (CDCl_3_, *δ* in ppm): 30.4 (CH_2_), 59.2 (CH_3_), 60.2 (CH), 69.1 (CH_2_), 70.1 (C_5_H_4_), 70.2 (C_5_H_5_), 70.3 (CH_2_), 70.4 (C_5_H_4_), 70.7 (CH_2_), 70.8 (CH_2_), 72.1 (CH_2_), 72.6 (C_5_H_4_), 72.7 (C_5_H_4_), 79.0 (*^i^*C-C_5_H_4_), 199.8 (CO). HRMS (ESI-TOF, *m*/*z*): calcd. for C_30_H_34_Fe_2_O_5_: 586.1105; found 586.1070 [M + H]^+^.

Crystal data for **9**: C_30_H_34_Fe_2_O_5_, M = 586.27 g mol^−1^, monoclinic, *P*2_1_/*c*, *λ* = 0.71073 Å, *a* = 29.244(2) Å, *b* = 9.6717(7) Å, *c* = 19.1333(11) Å, *β* = 102.630(6)°, *V* = 5280.7(6) Å^3^, *Z* = 8, *ρ*_calcd_ =1.475 Mg m^−3^, *μ* = 1.138 mm^−1^, *T* = 114.0(6) K, *θ* range 3.004–25.000°, 24,744 reflections collected, 9280 independent reflections (*R*_int_ = 0.0447), *R*1 = 0.0838, *w*R2 = 0.2179 (*I* > *2σ*(*I*)).

### 4.9. Synthesis of tris-(1,3-Diferrocenylpropane-1,3-dionato-κ^2^-O,O′)titanium(III) *(**10**)*

1,3-Diferrocenylpropane-1,3-dionato lithium (200 mg, 0.45 mmol) in 200 mL of anhydrous tetrahydrofuran was dropwise treated with TiCl_4_ (16 μL, 0.14 mmol) via a syringe at −80 °C. A green colored precipitate was formed immediately, and the reaction solution was stirred until it reached ambient temperature. The green precipitate was filtered off, washed thrice with 10 mL (each) of diethyl ether, and crystallization from toluene afforded a dark green solid of **10**. Yield: 29 mg (0.021 mmol, 14% based on 1,3-diferrocenylpropane-1,3-dionato lithium(I)).

C_69_H_57_Fe_6_O_6_Ti (M = 1365.12 g∙mol^−1^). Mp.: ≥240 °C decomposition. IR (KBr, in cm^−1^): 1618 (m), 1504 (s, ν*_C=O_*), 1501 (s, ν*_C=O_*), 1445 (m), 1381 (m), 1344 (m), 1331 (m), 1295 (m), 1250 (m), 1213 (w), 1107 (m), 1028 (m), 943 (m), 823 (m, π*_C–H_*, C_5_H_5_), 731 (m), 675 (m). HRMS (ESI-TOF, *m*/*z*): calcd. for C_69_H_57_Fe_6_O_6_Ti: 1364.9735; found 1364.9675 [M + H]^+^.

### 4.10. Synthesis of tris-(1,3-Diferrocenylpropane-1,3-dionato-κ^2^-O,O′)iron(III) *(**11**)*

A solution of [Fe(acac)_3_] (45 mg, 0.127 mmol) and **5** (167 mg, 0.38 mmol) in 15 mL of acetonitrile was refluxed for 3 h. The solvent was reduced to 4 mL, and the dark brown precipitate was filtered off and washed thrice with 10 mL (each) of diethyl ether to give **11**. Yield: 22 mg (0.016 mmol, 12% based on [Fe(acac)_3_]).

Anal. Calcd. for C_69_H_57_Fe_7_O_6_ (M = 1373.10 g∙mol^−1^): C 60.36, H 4.18; found: C 60.17, H 4.60. Mp.: ≥230 °C decomposition. IR (KBr, in cm^−1^): 3094 (m, ν*_C–H_*), 1536 (s, ν*_C=O_*), 1519 (s, ν*_C=O_*), 1462 (m), 1439 (m), 1404 (m), 1370 (m), 1350 (m), 1327 (m), 1300 (m), 1242 (m), 1210 (w), 1106 (m), 1024 (m), 947 (m), 820 (m, π*_C–H_*, C_5_H_5_), 790 (m), 688 (m), 565 (m, C_5_H_4_ ring tilt). 

### 4.11. Synthesis of bis-(1,3-Diferrocenylpropane-1,3-dionato-κ^2^-O,O′)difluoro-borane(III) *(**12**)*

To a solution of **5** (300 mg, 0.68 mmol) and 0.12 mL of diisopropyl amine (0.82 mmol) in 10 mL of dichloromethane was dropwise added 0.1 mL of [BF_3_^.^OEt_2_] (0.82 mmol) via a syringe at ambient temperature. The color changed immediately to purple. After 1 h of stirring at this temperature, all volatiles were removed under reduced pressure, and the crude product was purified by column chromatography (column size: 20 × 3 cm, silica) using dichloromethane as eluent. The 1st fraction contained **12**. Crystallization of **12** from dichloromethane at ambient temperature gave dark purple crystals. Yield: 36 mg (0.074 mmol, 11% based on **5**).

Anal. Calcd. for C_23_H_19_Fe_2_O_2_BF_2_ (M = 487.89 g∙mol^−1^): C 56.62, H 3.93; found: C 56.98, H 4.12. Mp.: ≥280 °C decomposition. IR (KBr, in cm^−1^): 3110 (w, ν*_C–H_*), 2925 (w), 2852 (w), 1541 (s, ν*_C=O_*), 1497 (s, ν*_C=O_*), 1456 (m), 1385 (m), 1369 (m), 1352 (m), 1322 (m), 1261 (m), 1218 (w), 1185 (w), 1146 (m), 1026 (m), 1107 (m), 1085 (m), 1062 (m), 1030 (m), 1019 (m), 916 (w), 823 (m, π*_C–H_*, C_5_H_5_), 807 (m), 739 (m), 685 (m), 510 (m, C_5_H_4_ ring tilt). ^1^H NMR (CDCl_3_, *δ* in ppm): 4.29 (s, 10H, C_5_H_5_), 4.76 (pt, ^3^*J*_H,H_ = 1.9 Hz, 4H, C_5_H_4_), 4.99 (pt, ^3^*J*_H,H_ = 1.9 Hz, 4H, C_5_H_4_), 6.10 (s, 1H, COCHCO). ^13^C{^1^H} NMR (CDCl_3_, *δ* in ppm): 69.8 (C_5_H_4_), 71.5 (C_5_H_5_), 74.5 (C_5_H_4_), 74.6 (*^i^*C_5_H_4_), 93.3 (CH), 185.5 (C=O). ^11^B{^1^H} NMR (CDCl_3_, *δ* in ppm): 0.94 ppm. 

Crystal data for **12**: C_23_H_19_BF_2_Fe_2_O_2_, *M* = 487.89 g mol^−1^, monoclinic, *P*2_1_/*c*, *λ* = 0.71073 Å, *a* = 12.0629(6) Å, *b* = 13.4134(5) Å, *c* = 12.3500(5) Å, β = 104.741(4)°, *V* = 1932.51(15) Å^3^, *Z* = 4, *ρ*_calcd_ = 1.677 Mg m^−3^, *μ* = 1.538 mm^−1^, *T* = 112.8(5) K, *θ* range 3.037–24.998°, 7728 reflections collected, 3393 independent reflections (*R*_int_ = 0.0344), *R*1 = 0.0334, *wR*2 = 0.0779 (*I* > *2σ*(*I*)).

### 4.12. Synthesis of 3,5-Diferrocenyl-1H-pyrazole *(**13**)*

Diferrocenyl diketone **5** (700 mg, 1.59 mmol) and 6 equiv. of 64% hydrazine hydrate (0.5 mL) in 10 mL of acetic acid were stirred for 12 h at 70 °C. After cooling the reaction mixture to ambient temperature, it was poured into water and neutralized with a 1 M solution of NaOH. The obtained solution was extracted four times with 30 mL (each) of dichloromethane, the combined organic phases were dried over MgSO_4_, and then, all volatiles were removed in vacuum. The crude product was adsorbed on silica and purified by column chromatography (column size: 20 × 3 cm, silica) using dichloromethane as eluent. The 3rd fraction contained **13**. After removing all volatiles under reduced pressure, compound **13** was obtained as an orange-red solid. Yield: 560 mg (1.28 mmol, 80% based on **5**).

Anal. Calcd. for C_23_H_20_Fe_2_N_2_ (M = 436.11 g∙mol^−1^): C 63.34, H 4.62, N 6.42; found: C 63.98, H 4.12, N 6.42. Mp.: ≥ 270 °C decomposition. IR (KBr, in cm^−1^): 3392 (m, ν*_N−H_*), 3213 (m, ν*_C–H_*), 3262 (m, ν*_C–H_*), 3093 (s, ν*_C–H_*), 3042 (m, ν*_C–H_*), 2926 (s), 2869 (m), 2760 (m), 1735 (w), 1637 (w), 1600 (s, ν*_C=N_*), 1550 (m), 1454 (w), 1414 (s, δ*_C-H_*), 1370 (w), 1280 (m), 1237 (w), 1168 (m), 1105 (s, ν*_C-N_*), 1085 (w), 1024 (m), 1001 (m), 980 (m), 879 (m), 813 (s, π*_C–H_*, C_5_H_5_), 713 (w), 538 (w), 510 (s, C_5_H_4_ ring tilt). ^1^H NMR (CDCl_3_, *δ* in ppm): 4.11 (s, 10H, C_5_H_5_), 4.31 (broad signal, 4H, C_5_H_4_), 4.66 (broad signal, 4H, C_5_H_4_), 6.33 (s, 1H, CH). ^13^C{^1^H} NMR (CDCl_3_, *δ* in ppm): 66.6 (C_5_H_4_), 68.9 (C_5_H_4_), 69.7 (C_5_H_5_), 100.2 (CH). HRMS (ESI-TOF, *m/z*): calcd. for C_23_H_20_Fe_2_N_2_: 436.0320; found 436.0344 [M]^+^.

Crystal data for **13**: C_92_H_82_Fe_8_N_8_O, M = 1762.45 g mol^−1^, triclinic, *P−*1, *λ* = 0.71073 Å, *a* = 12.7706(4) Å, *b* = 13.1931(5) Å, *c* = 25.2510(8) Å, α = 94.195(3)°, *β* = 90.511(2)°, *γ* = 117.837(3)°, *V* = 3747.5(2) Å^3^, *Z* = 2, *ρ*_calcd_ = 1.562 Mg m^−3^, *μ* = 1.559 mm^−1^, *T* = 114.8(3) K, *θ* range 3.339–24.996°, 28,847 reflections collected, 13,135 independent reflections (*R*_int_ = 0.0392), *R*1 = 0.0401, *wR*2 = 0.0852 (*I* > *2σ*(*I*)).

### 4.13. Synthesis of 1-Methyl-3,5-diferrocenyl-1H-pyrazole *(**14**)*

3,5-Diferrocenyl-1*H*-pyrazole (**13**) (64 mg, 0.15 mmol) dissolved in 5 mL of N*^i^*Pr_2_Et was stirred with two drops of nitrobenzene and 35 equiv. of dimethyl sulfate (0.5 mL) for 12 h at 70 °C. After cooling the reaction solution to ambient temperature and removing all volatile materials, the crude product was adsorbed on silica and purified by column chromatography (column size: 20 × 3 cm, silica) using a hexane–dichloromethane mixture of ratio 1:1 (*v*/*v*) as eluent. The 1st fraction contained the title complex. After removing all volatiles under reduced pressure, compound **14** was obtained as a yellow solid. Yield: 51 mg (0.11 mmol, 76% based on **13**).

C_24_H_22_Fe_2_N_2_ (M = 450.13 g∙mol^−1^). Mp.: ≥ 230 °C decomposition. IR (KBr, in cm^−1^): 3223 (s, ν*_C–H_*), 3176 (s, ν*_C–H_*), 3262 (s, ν*_C–H_*), 3094 (s, ν*_C–H_*), 2927 (s), 2855 (m), 1735 (w), 1654 (w), 1595 (m, ν*_C=N_*), 1550 (m), 1457 (w), 1412 (m, δ*_C-H_*), 1368 (w), 1274 (m), 1238 (w), 1171 (w), 1104 (s, ν*_C-N_*), 1085 (w), 1039 (m), 1030 (s), 1004 (m), 975 (w), 879 (s), 828 (m), 814 (s, π*_C–H_*, C_5_H_5_), 701 (w), 537 (w), 512 (s, C_5_H_4_ ring tilt). ^1^H NMR (CDCl_3_, *δ* in ppm): 3.97 (s, 3H, CH_3_), 4.10 (s, 5H, C_5_H_5_), 4.18 (s, 5H, C_5_H_5_), 4.27 (pt, ^3^*J*_H,H_ = 1.8 Hz, 2H, C_5_H_4_), 4.36 (pt, ^3^*J*_H,H_ = 1.9 Hz, 2H, C_5_H_4_), 4.53 (pt, ^3^*J*_H,H_ = 1.9 Hz, 2H, C_5_H_4_), 4.68 (pt, ^3^*J*_H,H_ = 1.8 Hz, 2H, C_5_H_4_), 6.32 (s, 1H, CH). HRMS (ESI-TOF, *m/z*): calcd. for C_24_H_22_Fe_2_N_2_: 450.0477; found 450.0478 [M]^+^.

### 4.14. Synthesis of 1-Phenyl-3,5-diferrocenyl-1H-pyrazole *(**15**)*

A mixture of compound **5** (200 mg, 0.454 mmol) and 4.4 equiv. of phenylhydrazine (0.2 mL) in 10 mL of acetic acid was stirred at 70 °C for 24 h. After cooling the reaction solution to ambient temperature, it was poured into water and neutralized with a 1 M solution of NaOH. The solution was extracted thrice with 40 mL of dichloromethane (each), the combined organic phases were dried over MgSO_4_, and then all volatiles were removed in vacuum. The crude product was adsorbed on silica and purified by column chromatography (column size: 20 × 3 cm, silica) using dichloromethane as eluent. The 2nd fraction contained the title compound. After removing all volatiles under reduced pressure, compound **15** was obtained as an orange solid. Orange crystals of **15** were formed by crystallization of **15** from a hexane–dichloromethane mixture of ratio 1:10 (*v*/*v*) at −20 °C. Yield: 63 mg (0.123 mmol, 27% based on **5**).

Anal. Calcd. for C_29_H_24_Fe_2_N_2_ (M = 512.20 g∙mol^−1^): C 68.00, H 4.72, N 5.47; found: C 67.60, H 4.69, 5.37. Mp.: 158°. IR (KBr, in cm^−1^): 3103 (m, ν*_C–H_*), 3095 (m, ν*_C–H_*), 3085 (s, ν*_C–H_*), 1593 (m, ν*_C=N_*), 1573 (m), 1543 (m), 1498 (s), 1449 (m), 1417 (s, δ*_C-H_*), 1393 (m), 1364 (m), 1352 (m), 1323 (m), 1226 (w), 1210 (w), 1180 (m), 1106 (s, ν*_C-N_*), 1095 (w), 1074 (m), 1028 (m), 999 (m), 980 (m), 969 (m), 911 (m), 882 (m), 873 (m), 829 (s), 816 (s, π*_C–H_*, C_5_H_5_), 799 (s), 759 (s), 697 (m), 684 (m), 650 (m), 579 (m), 528 (m), 513 (s, C_5_H_4_ ring tilt). ^1^H NMR (CDCl_3_, *δ* in ppm): 4.09 (s, 5H, C_5_H_5_), 4.14 (s, 5H, C_5_H_5_), 4.18 (broad signal, 2H, C_5_H_4_), 4.20 (broad signal, 2H, C_5_H_4_), 4.30 (broad signal, 2H, C_5_H_4_), 4.77 (broad signal, 2H, C_5_H_4_), 6.55 (s, 1H, CH), 7.40 (m, 5H, C_6_H_5_). ^13^C{^1^H} NMR (CDCl_3_, *δ* in ppm): 66.9 (C_5_H_4_), 68.7 (C_5_H_4_), 68.7 (C_5_H_4_), 69.7 (C_5_H_4_), 70.0 (C_5_H_5_), 75.1 (*^i^*C-C_5_H_4_), 78.5 (*^i^*C-C_5_H_4_), 104.3 (CH-C_3_N_2_), 126.6 (C_6_H_5_), 128.1 (C_6_H_5_), 129.0 (C_6_H_5_), 140.7 (*^i^*C C_6_H_5_), 142.5 (*^i^*C-C_3_N_2_), 151.2 (*^i^*C-C_3_N_2_). HRMS (ESI-TOF, *m/z*): calcd. for C_29_H_24_Fe_2_N_2_: 512.0634; found 512.0628 [M]^+^.

Crystal data for **15**: C_29_H_24_Fe_2_N_2_, M = 512.20 g mol^−1^, monoclinic, *P*2_1_/*n*, *λ* = 0.71073 Å, *a* = 12.7813(4) Å, *b* = 9.8440(3) Å, *c* = 17.3856(6) Å, *β* = 90.168(3)°, *V* = 2187.43(12) Å^3^, *Z* = 4, *ρ*_calcd_ = 1.555 Mg m^−3^, *μ* = 1.347 mm^−1^, *T* = 113.2(4) K, *θ* range 3.126–24.998°, 14328 reflections collected, 3843 independent reflections (*R*_int_ = 0.0365), *R*1 = 0.0444, *wR*2 = 0.1175 (*I* > *2σ*(*I*)).

## Figures and Tables

**Table 1:** Hydrogen bond and *T*-shaped *π*-interaction properties (Å/°) of **13**.

**Hydrogen Bridge-Bonds**		
D–H⋯A	D–A/Å	D–H⋯A/°
O1–H1O⋯N3	2.714(3)	173(4)
O1–H2O⋯N7	2.723(4)	169(4)
N2–H2A⋯O1	2.801(4)	165
N5–H5A⋯O1	2.732(3)	167
N4–H4⋯N6	3.088(4)	150
N8–H8A⋯N1	3.084(4)	164
***T*-shaped *π*-interactions**		
Ct(C_5_H_4_)⋯Ct(C_5_H_5_)	D/Å	α/°
C27–31⋯C14–18	4.741(3)	87.7(2)
C50–54⋯C73–77	4.651(3)	85.7(2)

The angle α is described by the intersection of the involved C_5_H_4_/C_5_H_5_ planes.

**Table 2:** Cyclic voltammetry data of **5**‒**7** and **9**‒**15**. All Fc potentials are given in mV. ^a.^

Compd.	*E*_1_°′ *^c^* (Δ*E*_p_) *^d^*	*E*_2_°′ *^c^* (Δ*E*_p_) *^d^*	*E*_3_°′ *^c^* (Δ*E*_p_) *^d^*	*E*_4_°′ *^c^* (Δ*E*_p_) *^d^*	*E*_5_°′ *^c^* (Δ*E*_p_) *^d^*	*E*_6_°′ *^c^* (Δ*E*_p_) *^d^*	Δ*E*°′ *^e^*
**5**	110 (60)	310 (88)					200
**6**	110 (60)	330 (60)					220
**7**	240 (60)	460 (74)					220
**9**	235 (60)	430 (70)					195
**10** ^b^	140 ^f^	260 ^f,g^		385 ^f^	485 ^f^	670 ^f^	120/125/100/185
**11**	100 ^f^	230 ^f^	305 ^f^	430 ^f^	560 ^f^	730 ^f^	130/75/125/130/170
**Al** ^h^	33 (62)	123 (62)	304 (72)	432 (56)	583 (62)	741 (58)	90/181/128/151/158
**12**	300 (62)	600 (64)					2145/300
**13**	−30 (60)	210 (64)					240
**14**	−30 (62)	210 (64)					240
**15**	−30 (65)	205 (66)					235

Potentials vs. FcH/FcH^+^ (FcH = Fe(η^5^-C_5_H_5_)_2_), scan rate 100 mV s^−1^ at a glassy-carbon electrode of 1.0 mmol·L^−1^ solutions in anhydrous dichloromethane containing 0.1 mol·L^−1^ of [NBu_4_][B(C_6_F_5_)_4_] as supporting electrolyte at 25 °C. (^a^) Reduction processes for **10** and **12** are: −985 mV (Δ*E*_p_ = 60 mV, **10**) and –1845 mV (Δ*E*_p_ = 64 mV, **12**); complex **11** did not show any reduction until −2200 mV. (^b^) An additional reversible redox process for Ti^IV^/Ti^III^ is found at −520 mV (Δ*E*_p_ = 64 mV) (Figure 9). (^c^) *E*_n_°′ = Formal potential of n Fc redox process. (^d^) Δ*E*_p_ = Difference between oxidation and reduction potentials. (^e^) Δ*E*°′ = Potential difference between the ferrocenyl-related redox processes. (^f^) Potentials from square-wave voltammogram (dotted line: step-height 25 mV, pulse-width 5 s, amplitude 5 mV) (Figure 9). (^g^) Two electron process. (^h^) Al = [Al(κ^2^*O*,*O*′-FcC(O)CHC(O)Fc)_3_], data taken from Ref. [41].

## Supplementary Materials

The Supplementary Materials are available online. Figures, Tables, and CIF files giving further (spectro)electrochemical spectra, NMR spectra, and crystallographic data. Crystallographic data of **5**, **7**, **9**, **12**, **13**, **15**, and **16** are also available from the Cambridge Crystallographic Database as file numbers CCDC 1548123 (**5**), 1548124 (**7**), 1548125 (**9**), 1548126 (**12**), 1548127 (**13**_4_⋯H_2_O), 1548128 (**15**), 1548129 (**16**). Figure S1: Ball-and-Stick model of the molecular structure of **13** showing the intramolecular *T*-shaped π interactions (green). Geometric properties (Å/°): Ct_C4–C8_⋯Ct_C55–C59_, d = 4.637(8), α = 85.6(6); Ct_C37–C41_⋯Ct_C78–C82_, d = 4.736(7), α = 87.8(6). Figure S2: Cyclic voltammogram (solid line: scan rate 100 mV s^−1^) and square-wave voltammograms (dotted line: step-height 25 mV, pulse-width 5 s, amplitude 5 mV) of **16** in dichloromethane solution (1.0 mmol ·L^−1^) at 25 °C measured with a glassy carbon working electrode. Supporting electrolyte 0.1 mol·L^−1^ of [NBu_4_][B(C_6_F_5_)_4_]). Figure S3: Cyclic voltammogram (scan rate 100 mV s^−1^) of 11 in dichloromethane solution (1.0 mmol·L^−1^) at 25 °C measured with a glassy carbon working electrode. Supporting electrolyte 0.1 mol·L^−1^ of [NBu_4_][B(C_6_F_5_)_4_]). * Impurities in the electrolyte. Figure S4: UV‒Vis/NIR spectra of **5** in a dichloromethane solution (2.0 mmol·L^−1^) at rising potentials vs. Ag/AgCl at 25 °C; supporting electrolyte 0.1 mol·L^−1^ of [NBu_4_][B(C_6_F_5_)_4_]. Arrows indicate an increase, decrease or shift of absorptions. Figure S5: UV‒Vis/NIR spectra of **6** in a dichloromethane solution (2.0 mmol·L^−1^) at rising potentials vs. Ag/AgCl at 25 °C; supporting electrolyte 0.1 mol·L^−1^ of [NBu_4_][B(C_6_F_5_)_4_]. Arrows indicate an increase, decrease or shift of absorptions. Figure S6: Deconvolution of NIR absorption of [**12**]+ using Gaussian shaped bands. Table S1: Geometric properties (Å/°) of the ferrocenyls in **5**, **7**, **9**, **12**, **13** and **15**. Table S2: Plane Intersections (°) of C_5_H_4_, C_3_ and phenyl motifs in β-diketones, pyrazols and phenyls.
