# Supplementary material for: Synthesis, Characterization, and Electrochemistry of Diferrocenyl β-Diketones, -Diketonates, and Pyrazoles
Source: Molecules. 2020 Sep 29;25(19):4476. doi: 10.3390/molecules25194476 (PMC7583057; doi:10.3390/molecules25194476)

Synthesis, Characterization and Electrochemistry of Diferrocenyl *β*-Diketones, -Diketonates and Pyrazoles

Steve W. Lehrich, Julia Mahrholdt, Marcus Korb, Alexander Hildebrandt, Jannie C. Swarts and Heinrich Lang

SUPPORTING INFORMATION

Table of Contents **Page**

Solid State Structures

Table S1. Geometric Properties of the ferrocenyls in **5**, **7**, **9**, **12**, **13** and **15**. S2

Table S2. Plane intersections. S2

Fig. S1. Cyclic and square-wave voltammogram of **16**. S3

Electrochemistry

Fig. S2. Cyclic and square-wave voltammogram of **16**. S3

Fig. S3. Cyclic voltammogram of **11**. S3

Fig. S4. UV–Vis/NIR spectra of **5**. S4

Fig. S5. UV–Vis/NIR spectra of **6**. S4

Fig. S6. Deconvolution of NIR absorption of [**12**]$^{+}$. S5

^{1}H, ^{13}C{^{1}H} and ^{11}B{^{1}H} NMR spectra

^{1}H and ^{13}C{^{1}H} NMR spectra of **7**. S6

^{1}H and ^{13}C{^{1}H} NMR spectra of **9**. S7

^{1}H and ^{13}C{^{1}H} and ^{11}B{^{1}H} NMR spectra of **12**. S8

^{1}H and ^{13}C{^{1}H} NMR spectra of **13**. S9

^{1}H NMR spectrum of **14**. S10

^{1}H and ^{13}C{^{1}H} NMR spectra of **15**. S11

Table S1. Geometric properties (Å/°) of the ferrocenyls in **5**, **7**, **9**, **12**, **13** and **15**.

	**5**	**7**	**9**	**12**	**13**	**15**
	Bond distances (Å)					
Fe–Ct(C_5H_4)	1.6452(11)	1.6455(7)	1.6392(7)	1.6527(4)	1.6508(4), 1.6430(4)	1.6560(4)
	1.6468(11)	1.6457(7)	1.6399(8)	1.6455(3)	1.6444(4), 1.6436(4)	1.6490(4)
			1.6366(7)		1.6497(4), 1.6536(5)	
			1.6405(8)		1.6430(4), 1.6479(4)	
Fe–Ct(C_5H_5)	1.6525(11)	1.6568(7)	1.6498(7)	1.6616(4)	1.6589(4), 1.6466(4)	1.6597(4)
	1.6527(11)	1.6509(7)	1.6495(8)	1.6559(3)	1.6453(4), 1.6505(4)	1.6490(4)
			1.6501(7)		1.6470(4), 1.6496(5)	
			1.6550(8)		1.6451(4), 1.6488(4)	
	Tilt angles (°)					
Ct–Fe–Ct	178.35(8)	175.66(5)	176.72(6)	178.79(2)	178.74(3), 178,01(3)	177.16(3)
	179.60(9)	177.76(5)	177.64(6)	178.97(2)	177.82(3), 177.69(3)	178.50(3)
			176.31(6)		178.70(3), 178.88(3)	
			175.83(6)		178.40(3), 177.72(3)	
	Torsion Angles (°)					
$C_5H_4 \cdots C_5H_5$	1.2(6)	7.3(3)	7.2(4)	16.65(17)	18.0(2), 16.2(2)	10.4(2)
	12.0(7)	2.1(4)	4.3(5)	6.05(17)	22.4(2), 1.8(2)	0.11(19)
			2.2(4)	2.40(18)	12.2(2), 16.4(3)	
			3.9(5)		2.5(2), 15.1(2)	

Ct = Centroids of the respective fragment.

Table S2. Plane Intersections (°) of C_5H_4 , C_3 and phenyl motifs in β-diketones, pyrazols and phenyls.

	**5**	**7**	**9**	**12**	**13**	**15**	**16**[a)]
$C_5H_4 \cdots C_3$	10.6(10)	5.8(8)	2.0(4)	6.9(3)			19.9(5)
	23.1(8)	1.2(8)	4.6(7)	8.3(3)			12.7(5)
			1.6(4)				19.7(5)
			9.0(9)				17.0(5)
$C_5H_4 \cdots$Pz					14.47(7)/ 18.18(10)	45.65(13)	
					11.62(13)/ 14.19(8)	36.22(11)	
Pz···Pz					68.01(12)/69.17(13)		
					15.24(5)/14.89(15)		
Pz···*N*Ph						25.67(14)	

All rms deviations of the respective planes are below 0.007. Pz = Pyrazol ring. a) Ph rings instead of C_5H_4 groups.

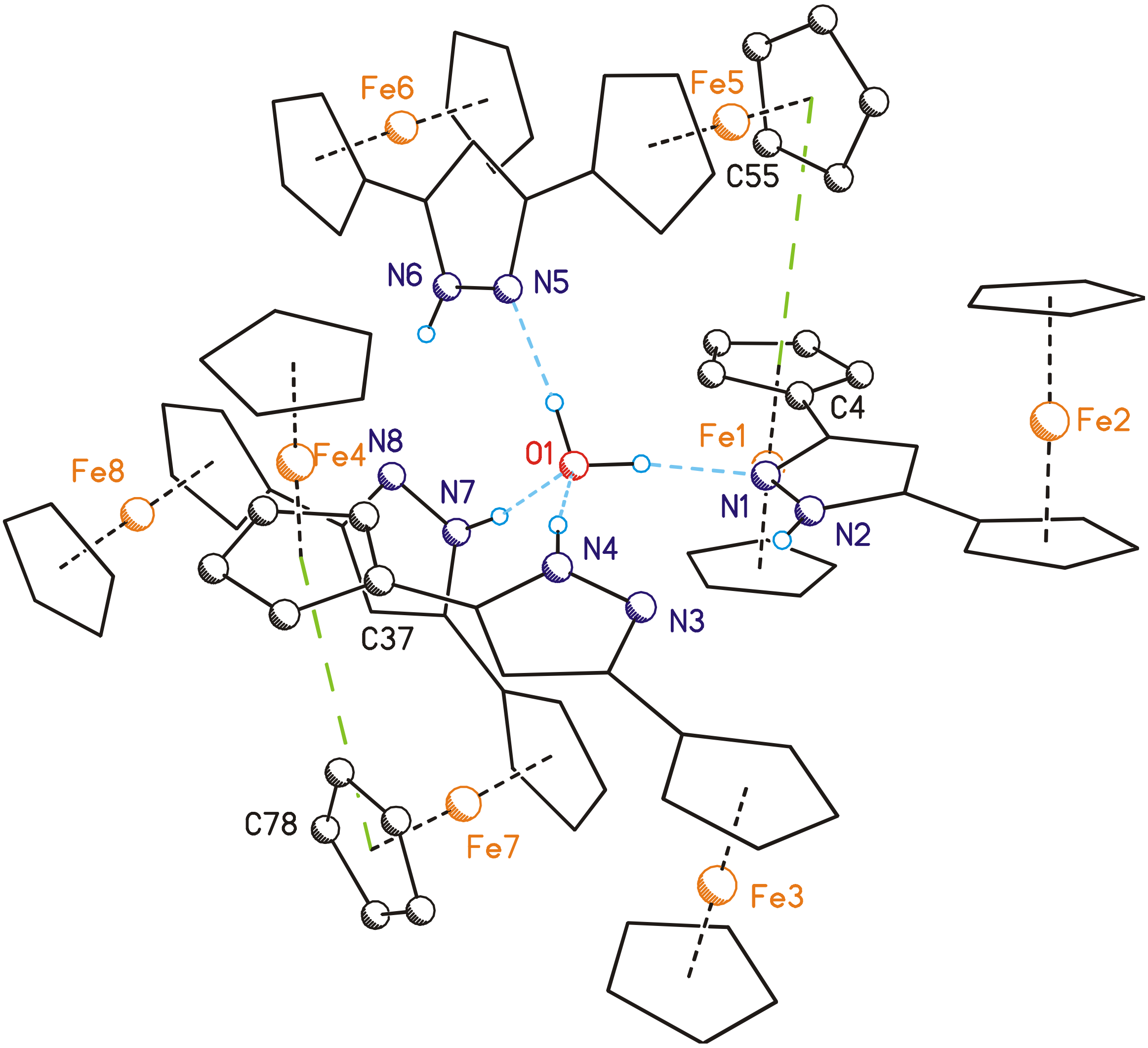

Fig. S1. Ball-and-Stick model of the molecular structure of **13** showing the intramolecular *T*-shaped π interactions (green). Geometric properties (Å/°): $Ct_{C4–C8}$··· $Ct_{C55–C59}$, d = 4.637(8), α = 85.6(6); $Ct_{C37–C41}$··· $Ct_{C78–C82}$, d = 4.736(7), α = 87.8(6).

Electrochemistry

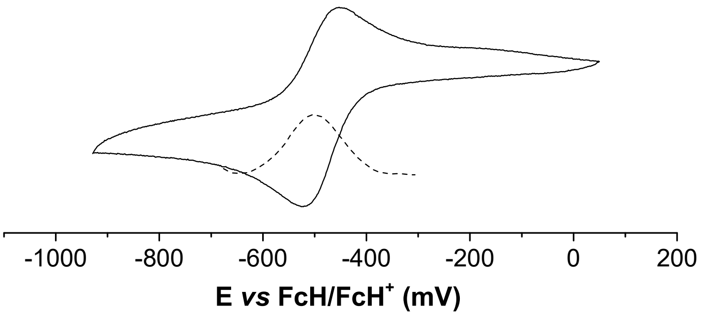

Fig. S2. Cyclic voltammogram (solid line: scan rate 100 mV s^{-1}) and square-wave voltammograms (dotted line: step-height 25 mV, pulse-width 5 s, amplitude 5 mV) of **16** in dichloromethane solution (1.0 mmol L^{-1}) at 25 °C measured with a glassy carbon working electrode. Supporting electrolyte 0.1 mol L^{-1} of $[NBu_4][B(C_6F_5)_4]$).

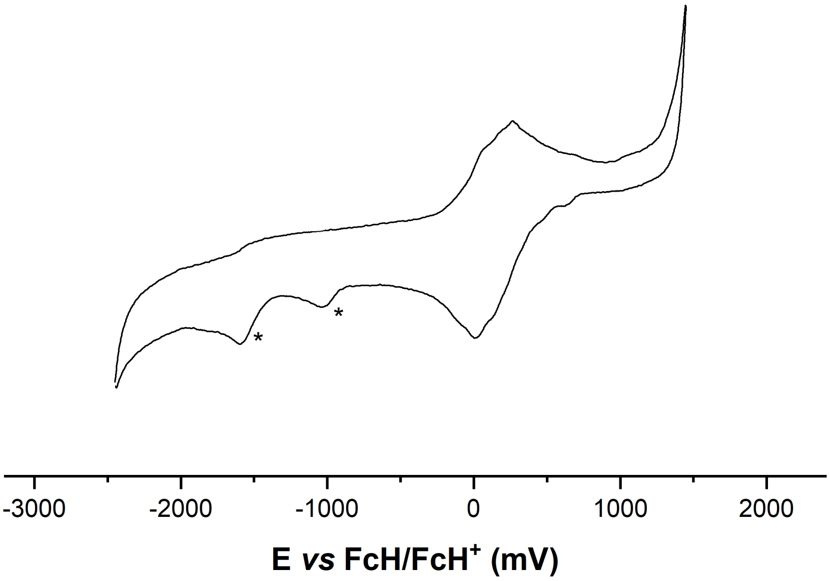

Fig. S3. Cyclic voltammogram (scan rate 100 mV s^{-1}) of **11** in dichloromethane solution (1.0 mmol L^{-1}) at 25 °C measured with a glassy carbon working electrode. Supporting electrolyte 0.1 mol L^{-1} of $[NBu_4][B(C_6F_5)_4]$). * Impurities in the electrolyte.

Spectroelectrochemistry

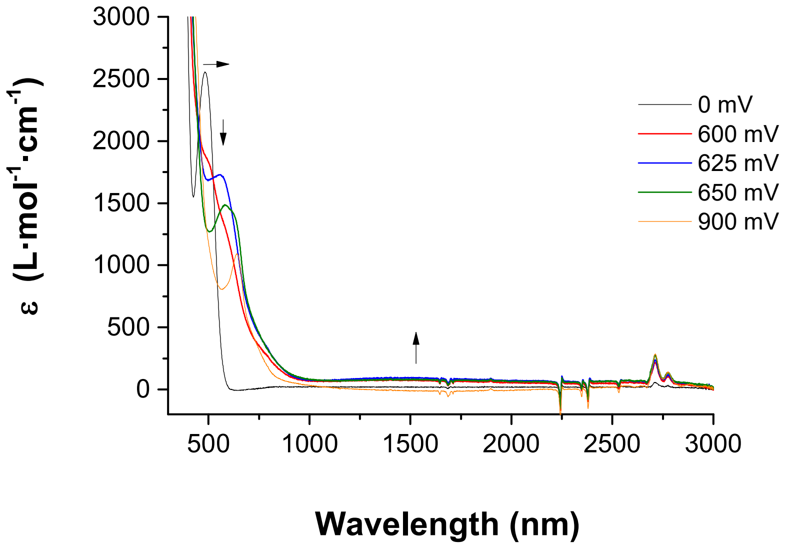

Fig. S4. UV–Vis/NIR spectra of **5** in a dichloromethane solution (2.0 mmol L^{-1}) at rising potentials *vs* Ag/AgCl at 25 °C; supporting electrolyte 0.1 mol L^{-1} of $[NBu_4][B(C_6F_5)_4]$. Arrows indicate an increase, decrease or shift of absorptions.

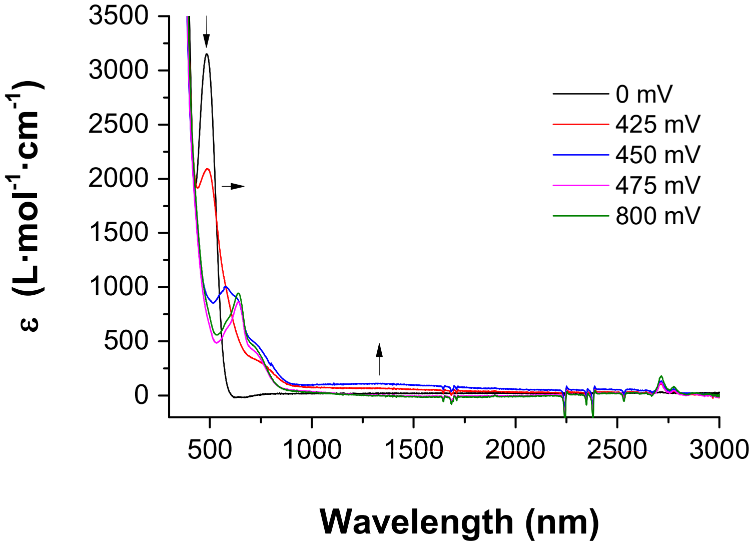

Fig. S5. UV–Vis/NIR spectra of **6** in a dichloromethane solution (2.0 mmol L^{-1}) at rising potentials *vs* Ag/AgCl at 25 °C; supporting electrolyte 0.1 mol L^{-1} of $[NBu_4][B(C_6F_5)_4]$. Arrows indicate an increase, decrease or shift of absorptions.

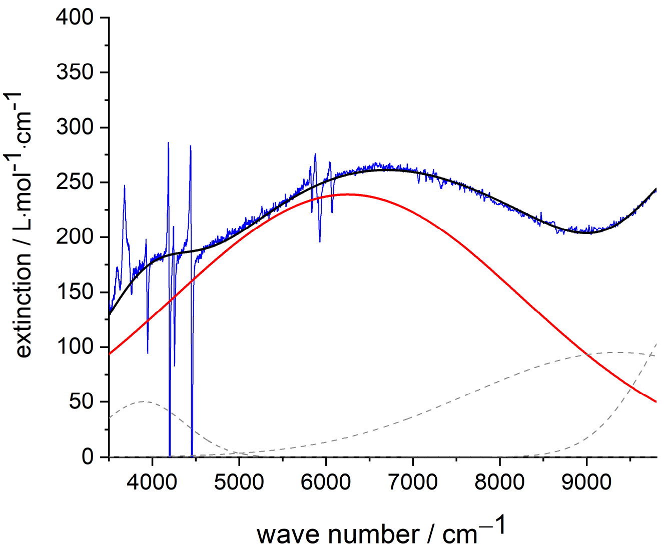

Fig. S6. Deconvolution of NIR absorption of [**12**]$^+$ using Gaussian shaped bands.

^{1}H, ^{13}C{^{1}H} and ^{11}B{^{1}H} NMR spectra

^{1}H and ^{13}C{^{1}H} NMR spectra of 7

Fc2C3O2Alkyl_1_2
PROTON CDCl3 {C:\Bruker\TOPSPIN} lest 54

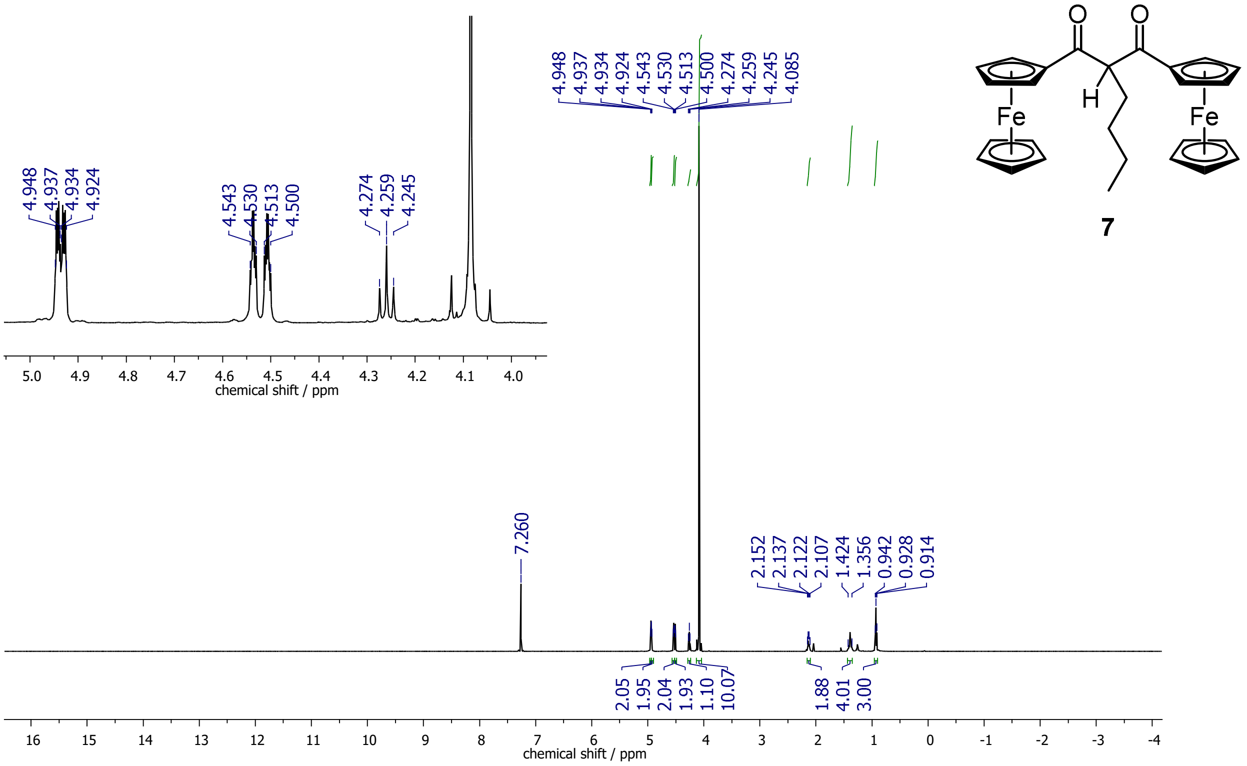

7

Fc2C3O2alkyl_1_2b
C13CPD CDCl3 {C:\Bruker\TOPSPIN} lest 29

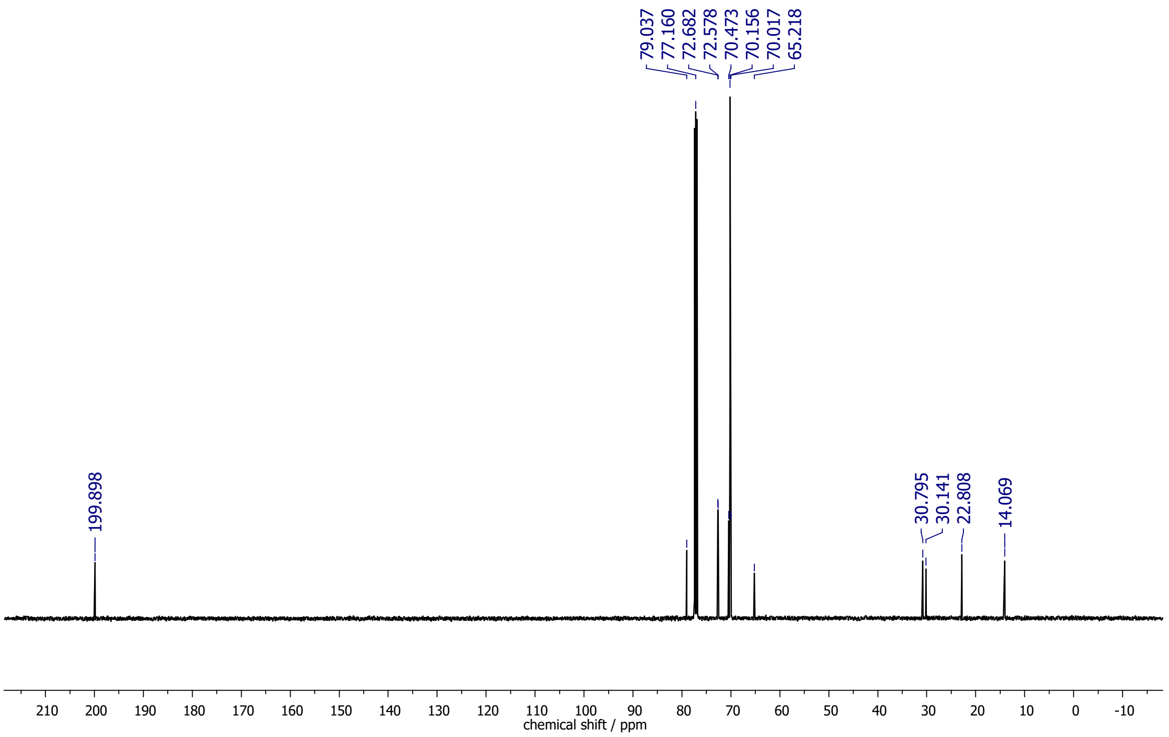

^{1}H and $^{13}C\{^{1}H\}$ NMR spectra of 9

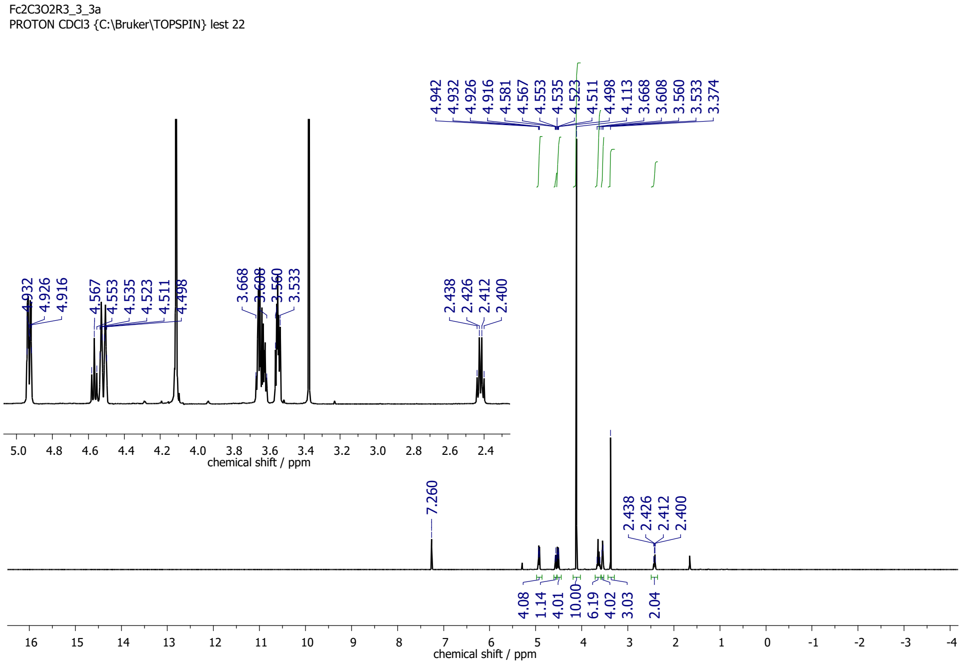

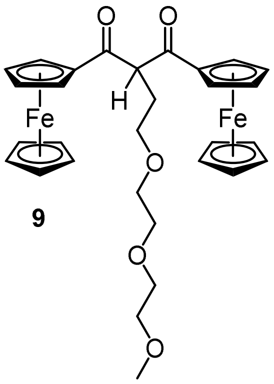

9

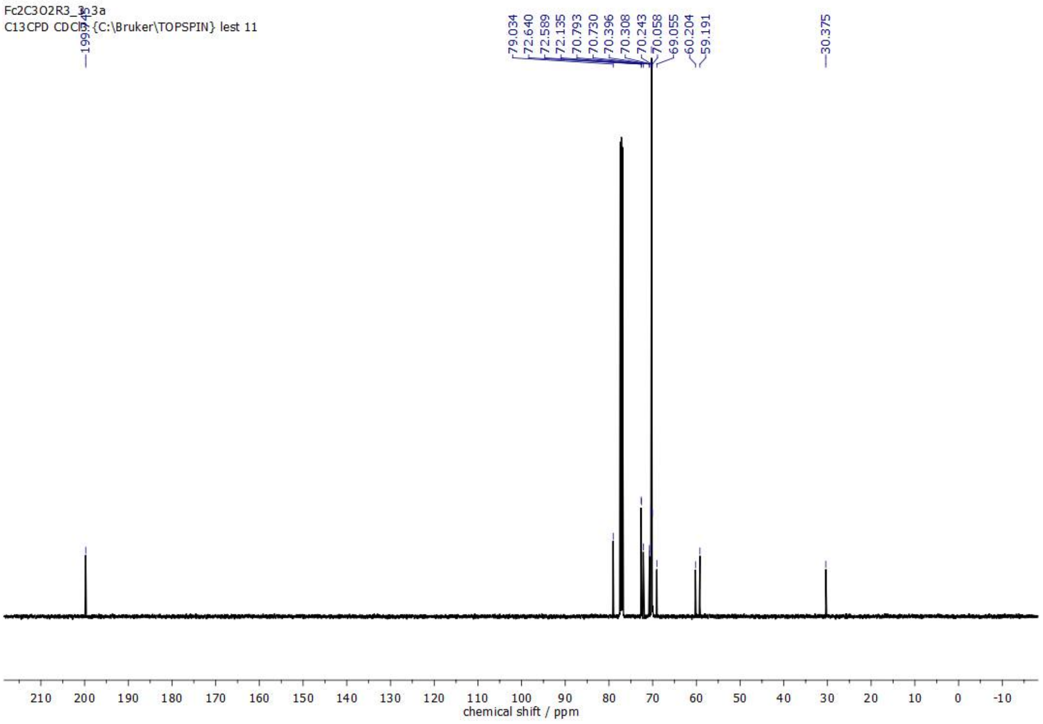

^{1}H, $^{13}C\{^{1}H\}$ and $^{11}B\{^{1}H\}$ NMR spectra of 12

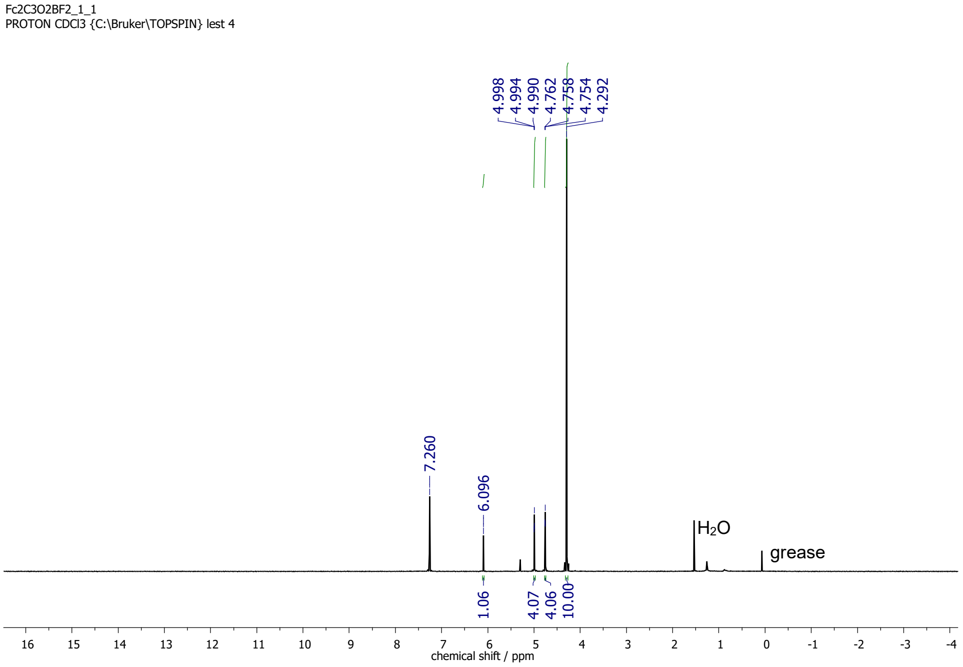

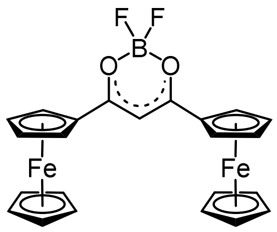

12

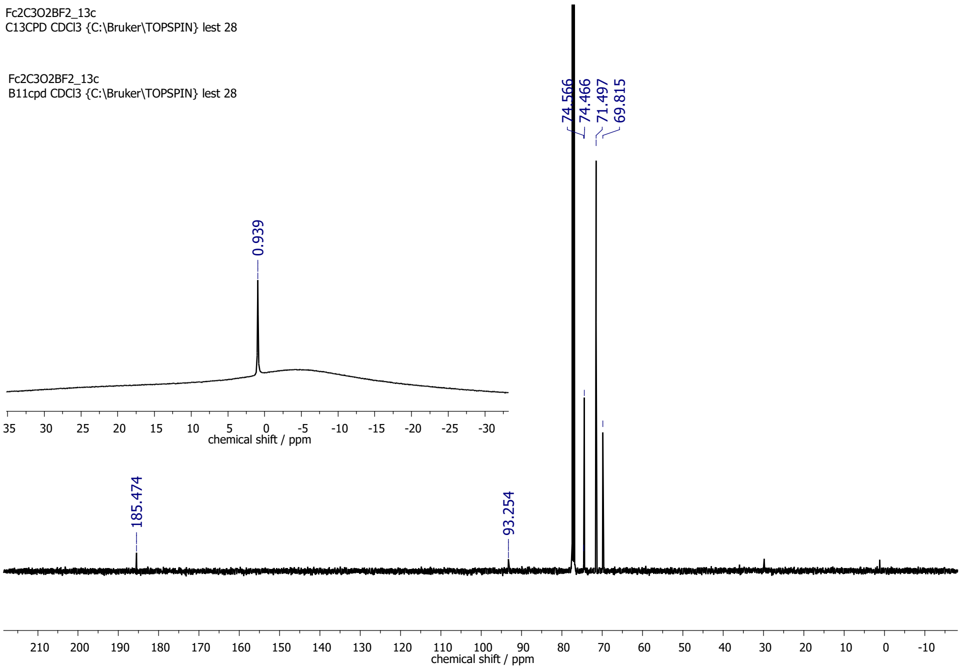

^{1}H and $^{13}C\{^{1}H\}$ NMR spectra of 13

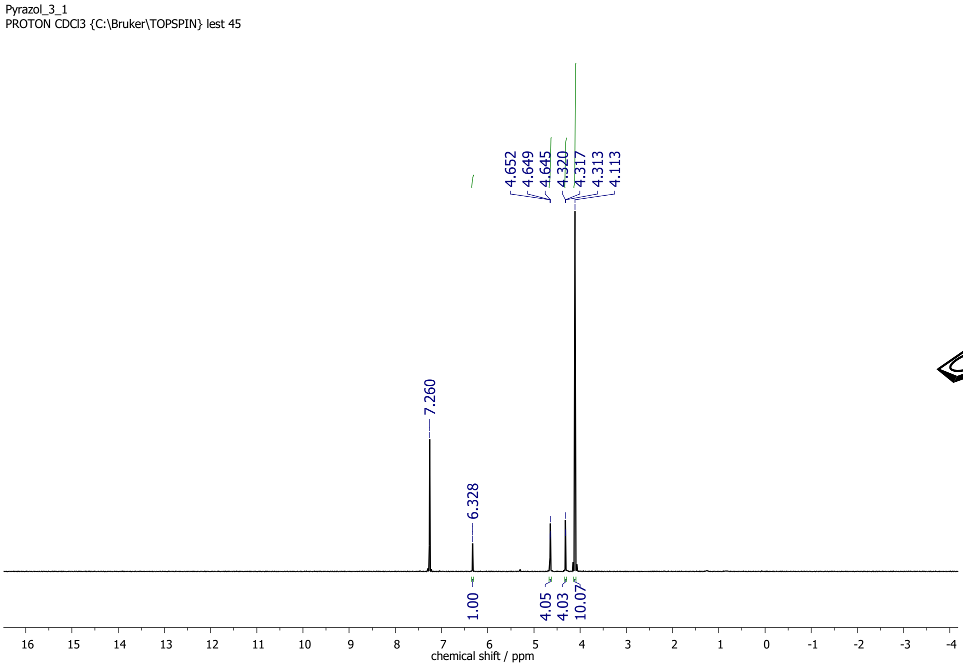

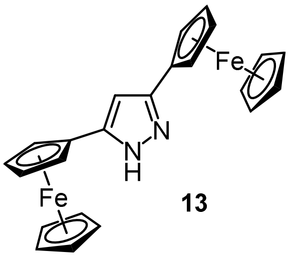

13

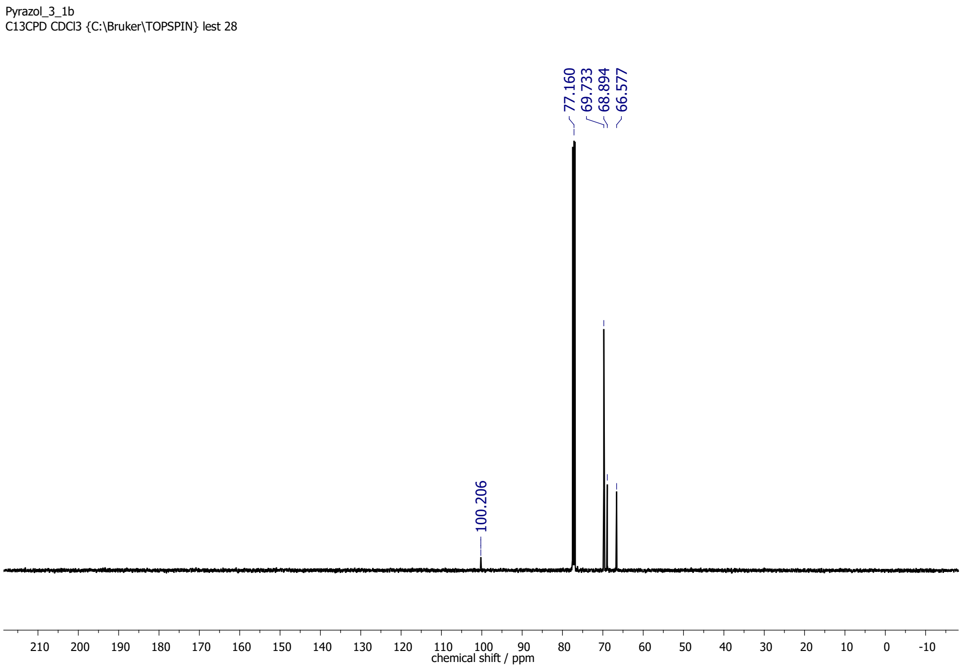

The *ipso*-carbon signals could not be found in the $^{13}C\{^{1}H\}$ NMR spectrum under the selected measurements conditions.

^{1}H NMR spectra of 14

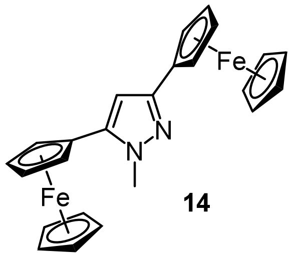

14

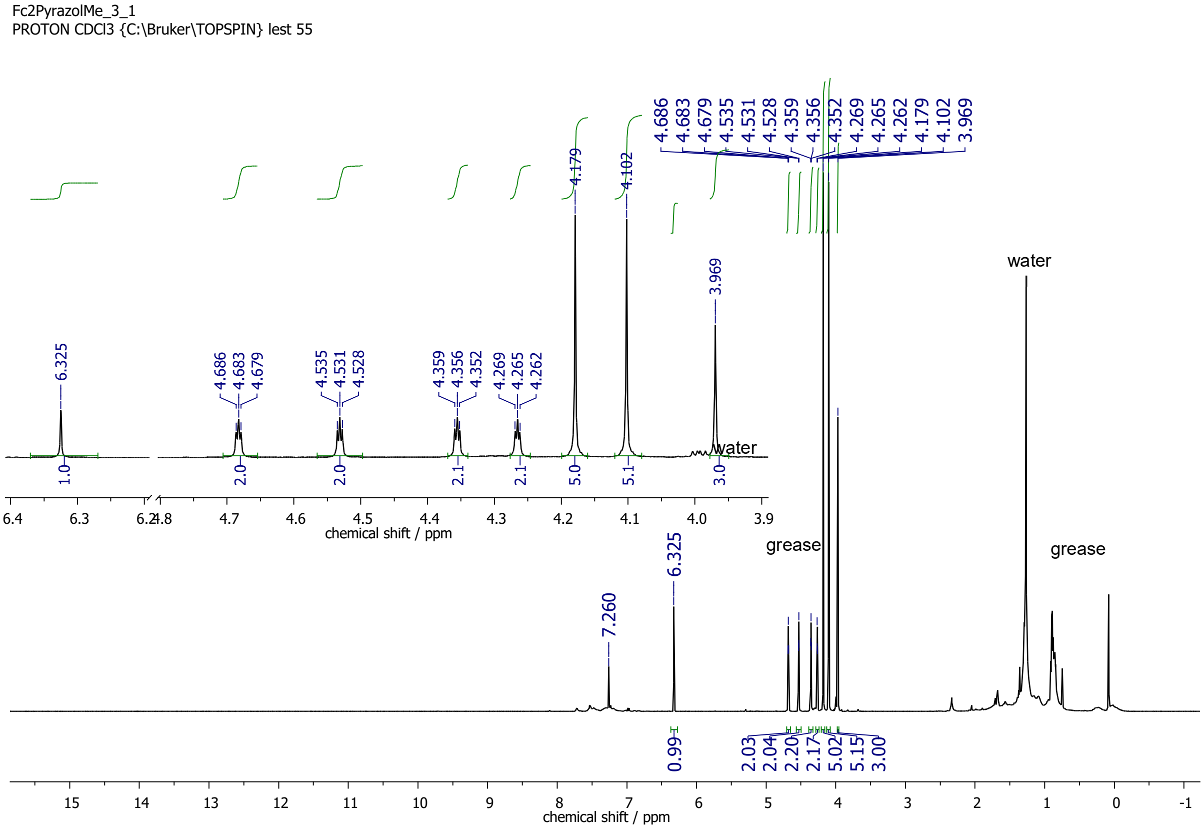

^{1}H and $^{13}C\{^{1}H\}$ NMR spectra of 15

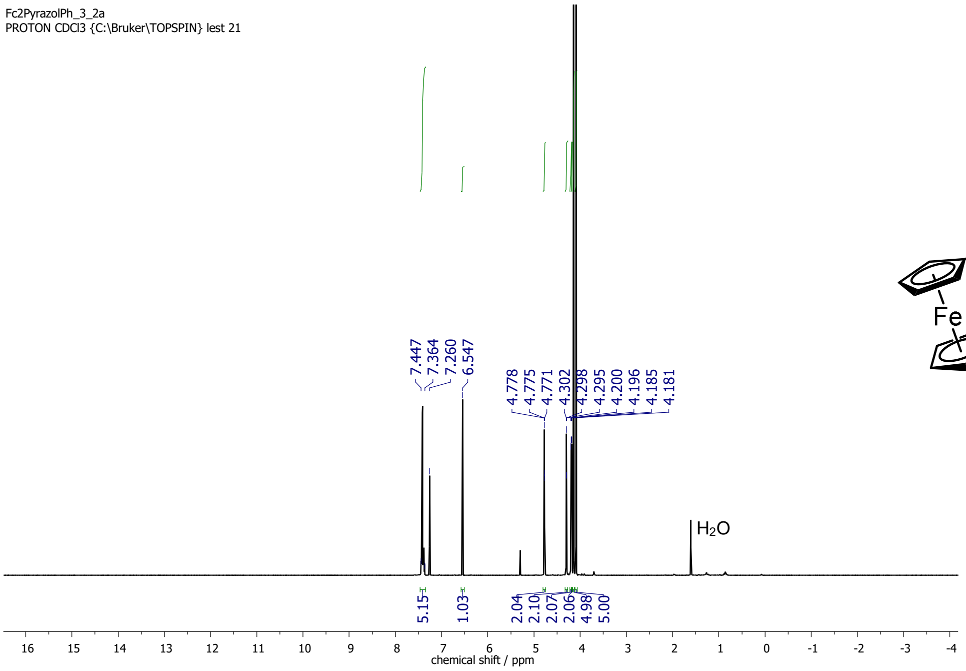

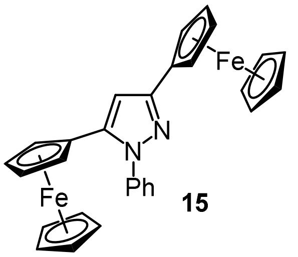

15

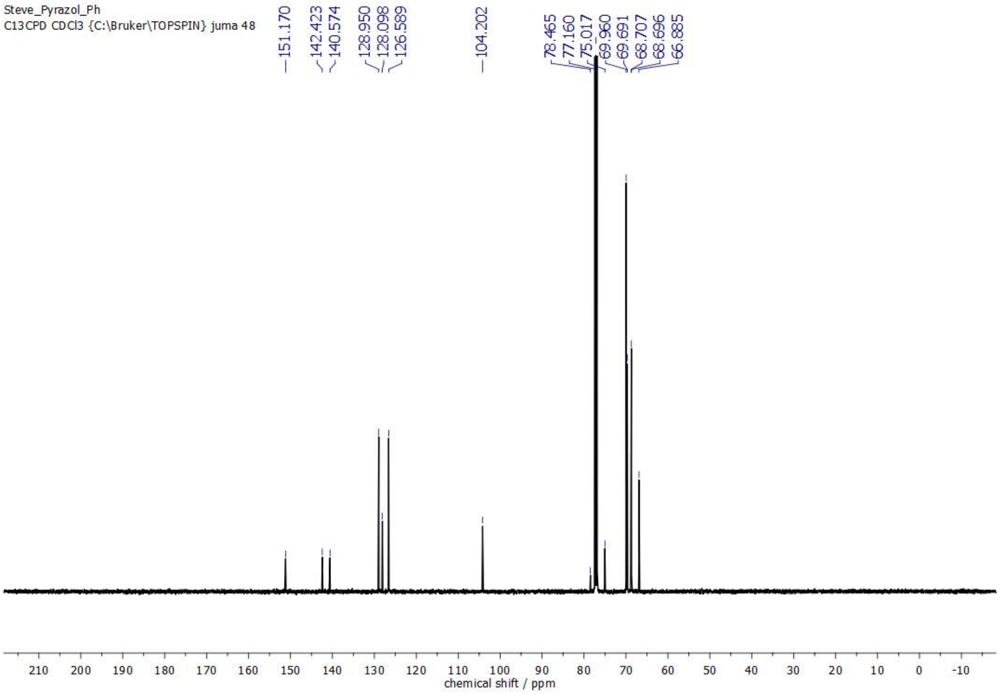

Supplement: Supplementary file 1 [file molecules-25-04476-s001.pdf]
